# *Staphylococcus aureus* ST59: Concurrent but Separate Evolution of North American and East Asian Lineages

**DOI:** 10.3389/fmicb.2021.631845

**Published:** 2021-02-10

**Authors:** Jo-Ann McClure, Sahreena Lakhundi, Amani Niazy, George Dong, Osahon Obasuyi, Paul Gordon, Sidong Chen, John M. Conly, Kunyan Zhang

**Affiliations:** ^1^Centre for Antimicrobial Resistance, Alberta Health Services/Alberta Precision Laboratories/University of Calgary, Calgary, AB, Canada; ^2^Department of Pathology & Laboratory Medicine, University of Calgary, Calgary, AB, Canada; ^3^Centre for Health Genomics and Informatics, University of Calgary, Calgary, AB, Canada; ^4^Department of Epidemiology and Health Statistics, Guangdong Pharmaceutical University, Guangzhou, China; ^5^Department of Microbiology, Immunology and Infectious Diseases, University of Calgary, Calgary, AB, Canada; ^6^Department of Medicine, University of Calgary, Calgary, AB, Canada; ^7^The Calvin, Phoebe and Joan Snyder Institute for Chronic Diseases, University of Calgary, Calgary, AB, Canada

**Keywords:** *Staphylococcus aureus*, multilocus sequence type ST59, whole genome sequences, phylogeny, evolution, mobile genetic element, mobile element structure, virulence

## Abstract

Despite initially being described in North America, *Staphylococcus aureus* (SA) sequence type ST59 is the most commonly isolated sequence type in Eastern Asia. The origins and evolution of this strain type remains unclear and therefore we gathered a collection of ST59 isolates from Canada and mainland China for a detailed genetic analysis of the lineage. Bayesian inference phylogenomic analysis of our isolates, along with previously published ST59 sequences indicated that the lineage could be divided into 6 distinct subgroups (WGS-1 thorough 6), each having distinct molecular characteristics. Analysis also demonstrated the concurrent but separate evolution of North American and East Asian lineages, as well as the extensive diversification of the East Asian lineage. The presence of a mobile element structure (MES) was found to be the major difference between these two continental lineages, absent in all North American isolates, and present in all East Asian ones. Other mobile genetic elements, such as the Immune Evasion Complex (IEC), Panton Valentine Leukocidin (PVL), and Staphylococcal Cassette Chromosome *mec* (SCC*mec*), showed significant variability within each sub-group and likely represents local selective pressures rather than major characteristics defining the groups. Our analysis also demonstrated the existence of a more ancient ST59 sub-lineage from North America, which was MES negative and contained some of the earliest reported ST59 isolates. Combined with the existence of a MES negative isolate from Taiwan, predicted to have appeared prior to diversification of the East Asian lineages, these results hint at the possibility of a North American origin for the lineage, which gained hold in Eastern Asia following acquisition of MES, and subsequently diversified.

## Introduction

Methicillin-resistant *Staphylococcus aureus* (MRSA) is an important human pathogen that causes a wide variety of infections ranging from skin and soft tissue infections to endocarditis and sepsis (Diekema et al., 2001). MRSA is a leading cause of hospital-acquired (HA-) infections worldwide (Pfaller et al., 1999; Fluit et al., 2001; Hoban et al., 2003), but has also become a significant cause of community-associated (CA-) infections in healthy individuals with no healthcare-associated risk factors (Begier et al., 2004; Harbarth et al., 2005; Ma et al., 2005; Vourli et al., 2005; Gilbert et al., 2006; Henderson and Nimmo, 2018; Lakhundi and Zhang, 2018). Genetic analysis using multilocus sequence typing (MLST) has revealed that community-associated MRSA (CA-MRSA) can be divided into five major lineages, including sequence types ST1, ST8, ST30, ST59, and ST80, however, they differ in their geographic dominance (Mediavilla et al., 2012; Chen and Huang, 2014). While MRSA sequence type ST8 (PFGE type USA300) has been the predominant CA-MRSA isolated in North America and some parts of Europe (Monecke et al., 2011), ST59 represents the most prevalent sequence type isolated in Eastern Asia, representing the major cause of skin and soft tissue infections, as well as being described as a commensal colonizer (Takano et al., 2007; Chen et al., 2013). The lineage is responsible for 56% of pediatric CA-MRSA cases in Taiwan (Huang et al., 2008; Chuang and Huang, 2013), and an increasing number of cases in mainland China (Li et al., 2018; Liang et al., 2019). While ST59 is also reported in other parts of the world, such as Europe and North America, it is not as commonly encountered in those regions.

Despite its lack of dominance in North America, ST59 was first reported in San Francisco in the early 2000s, classified as PFGE type USA1000, found to carry the Staphylococcal Cassette Chromosome *mec* (SCC*mec*) type IV, and predominantly negative for Panton-Valentine Leukocidin (PVL) gene carriage (Enright et al., 2002; Vandenesch et al., 2003; Diep et al., 2004). Shortly after being described in North America, ST59 was reported in Taiwan, with all isolates carrying PVL and a new SCC*mec* type, later characterized as type Vt (Wang et al., 2004; Boyle-Vavra et al., 2005). Reports of ST59 in mainland China followed (Ho et al., 2007).

The origin and spread of ST59 has been a topic of controversy, with some suggesting it originated in North America and spread to Asia, while others suggest it originated in Taiwan and spread from there (Vandenesch et al., 2003; Tristan et al., 2007; DeLeo et al., 2010; Hsu et al., 2010). Studies on the evolution of ST59 within Asia have noted that the lineage can be divided into 2 major clones, the Taiwan clone and the Asia-Pacific clone (Huang et al., 2008), with observations that the Taiwan clone can cause severe infections and carries the PVL-encoding phage Sa2, while the Asia-Pacific clone is typically commensal and carries the Sa3 phage with staphylokinase (Chen et al., 2013). Other studies have also examined the evolution and spread of ST59 within Taiwan and mainland China, describing sub clades within the regions differing in their carriage of mobile genetic elements related to multidrug resistance, immune evasion complex genes and SCC*mec* (Hung et al., 2012; Pang et al., 2020). One of those mobile genetic elements is the Mobile Element Structure (MES), responsible for multidrug resistance and carrying genes conferring resistance to erythromycin, clindamycin, kanamycin, streptomycin, and chloramphenicol (Hung et al., 2012). Acquisition of MES is believed to have been the precipitating event that allowed expansion of ST59 in Eastern Asia, with different variations of the element present in each region. A second mobile genetic element is the immune Evasion Complex (IEC), which carries genes such as *chp* (encoding the chemotaxis inhibitory protein), *scn* (encoding staphylococcal complement inhibitor) and *sak* (encoding staphylokinase), and is responsible for protecting the bacteria from the host’s immune response. Differing combinations of the IEC genes are suggested to be present in the differing lineages of ST59, and can be carried on ϕSa3 as in the Asian-Pacific clone, or on νSaβ as in the Taiwan clone (Hung et al., 2016). Finally, a large study encompassing more widespread global isolates determined that, unlike the theory that the lineage evolved in either North America or Asia and then spread, distinct clades of ST59 emerged concurrently in East Asia and the United States, occupying distinct geographical niches (Ward et al., 2016). Direct transfer of strains between the regions was not observed, but both clades subsequently spread to Australia and Europe (Ward et al., 2016).

Despite what is known, there still exist questions regarding the origin and spread of ST59. In particular, it is unclear why the lineage has become dominant in Asia while remaining relatively uncommon in North America, and what genetic or other differences account for this phenomenon. With the goal of further understanding the evolution of MRSA-ST59, we have gathered a collection of isolates from Canada and Guangdong China for detailed molecular analysis. Whole genome sequencing was done to determine if specific genomic differences exist between North American and Asian lineages which could account for their differential dominance. Whole genome phylogenetic analysis was also done with our isolates, combined with previously described global ST59 strains, to provide a more complete evolutionary picture of this important lineage.

## Materials and Methods

### Bacterial Isolates

Fifty-eight *S. aureus* ST59 were obtained between 1992 and 2014, from patients attending local clinics in Calgary, Alberta, Canada. An additional 162 *S. aureus* ST59 were collected from public community school students or hospital patients during a MSSA/MRSA epidemiological prevalence survey from February to April 2010 in Guangzhou, Guangdong, China. One isolate (G097) from Geneva and three isolates (CAN20, CAN49, and ST16) from Malaysia were present in our strain collection and added to the study. Canadian PFGE reference strains CMRSA1-10 were provided by the National Microbiology Laboratory, Health Canada, Winnipeg, Manitoba, Canada.

### Isolate Molecular Characterization

Staphylococcal isolates were fingerprinted by pulsed field gel electrophoresis (PFGE) after digestion with Smal (Mulvey et al., 2001). Fingerprints were digitized and analyzed with BioNumerics Ver. 6.6 (Applied Maths, Sint-Martens-Latem, Belgium) using the unweighted pair-group method using arithmetic averages (UPGMA), with relatedness calculated based on the Dice coefficient, with a position tolerance of 1.5 and an optimization of 0. A multiplex PCR assay was used to distinguish MRSA from MSSA while simultaneously detecting the PVL genes (Zhang et al., 2008). Isolates were further characterized with *spa* typing (Harmsen et al., 2003), MLST (Enright et al., 2000), and SCC*mec* typing (McClure et al., 2010; Zhang et al., 2012).

Genome sequencing was done on representative isolates with Illumina MiSeq technology (Illumina Inc., San Diego, CA, United States), whereby at least one representative was selected from each major branch in the PFGE tree, with major branches defined as sharing a 78% or greater Dice coefficient of correlation (DCC). Long sequence reads for 5 randomly selected isolates were obtained with Pacific Biosciences (PacBio) RSII sequencing technology. *Denovo* assembly was done on all Illumina reads using DNASTAR Lasergene v15.1 (Madison, WI, United States), while PacBio reads were assembled by the McGill University Génome Québec Innovation Centre using the HGAP workflow, whereby short reads were used to correct long read, which were subsequently assembled (Montréal, Québec, Canada) (Chin et al., 2013). ST59 sequencing reads, for strains described by Ward et al. (2016), were downloaded from the European Nucleotide Archive repository and *de novo* assembly done as described. Contigs for strains described by Pang et al. (2020) were downloaded from the National Center for Biotechnology Information (NCBI). HZW450 (CP020741), M013 (CP003166), SA957 (CP003603), SA268 (CP006630), and SA40 (CP003604) sequences were obtained from the National Center for Biotechnology Information (NCBI). Short and long sequencing reads (when applicable) have been deposited in NCBI under Bioproject number PRJNA663694.

Vector NTI Advance v11.5.2 (Invitrogen) was used to search for and identify the Immune Evasion Complex (IEC) genes, as well as Mobile Element Structure (MES) genes. For downloaded sequences with no isolate physically available, *de novo* assembly was done and then the PVL genes identified with Virulence finder 2.0 (Joensen et al., 2014), *spa* types determined with spaTyper v. 1.0 (Bartels et al., 2014) and SCC*mec* determined with SCCmecFinder v. 1.2 (Kaya et al., 2018). The genes (including mobile element genes) were also searched for and identified within the *de novo* assembled sequence using Vector NTI Advance, for confirmation. Genetic relatedness was calculated by *in silico* DNA–DNA hybridization using the online software GGDC 2.1, formula 2 as recommended (Meier-Kolthoff et al., 2013).

### Evolutionary Analysis

The genomes of 174 ST59 strains plus outgroup genomes COL, Newman, and N315 were aligned to RefSeq ST59 strain SA40 using snippy^1^. The default minimum coverage parameter value of 10 was used for threshold variant calling. The SNP-substituted genomes were run through Gubbins (Croucher et al., 2015) using a hybrid tree model until convergence, to identify putative recombination sites, using the three outgroups genomes for tree re-rooting. These putative recombination sites were masked in the alignment using the bedtools (Quinlan, 2014) maskfasta command, and the outgroup sequences removed before all subsequent analyses. SNP-sites (Page et al., 2016) were used to generate a monomorphic sites file. A NEXUS formatted file was generated with the following input data derived from the genomes: (1) the monomorphic sites file (using a DNA alphabet), and (2) a set of binary present/absent flags representing genomic insertions and deletions. These putative insertions and deletions were generated earlier by snippy. Bayesian inference phylogenomic trees were generated using BEAST2 (Bouckaert et al., 2014), run for 100M iterations (sampling frequency 1000) on this NEXUS input using a gamma site model and six partitions (six site models): codon position 1 SNPs, codon position 2 SNPs, codon position 3 SNPs, non-coding SNPs, coding insertions/deletions, and non-coding insertions/deletions. All six partitions used a 4-category gamma site model. All four SNP partitions shared a relaxed log normal clock model and each used an estimated Generalize Time Reversible substitution model similar to the analysis by Pang et al. (2020), while the insertions/deletions shared another relaxed log normal clock model and Mutation Death Model for substitution. The insertions and deletions in three PacBio assembled genomes (GD1409, GD1958, GD75) were masked due to observed likely high false positive rate. A Coalescent Bayesian Skyline model for priors was employed with default parameters.

Known tip dates were encoded for each sample, with the only unknown tip date (CAN20-Malaysia) modal estimate being 2005 (logP = −3) using it and all 173 dated genomes in 10M iterations of a gamma site model with an HKY substitution model and relaxed log normal clock.

Tree convergence was confirmed using BEAST’s Tracer 1.7.1 program (Suchard et al., 2018) using the recommended criterion (ESS > 200). TreeAnnotator was then used to identify the maximum clade credibility (MCC) tree using a 10% burn-in. The resulting tree was visualized using BEAST’s FigTree 1.4.4.

### Virulence Assessment Using the *Caenorhabditis elegans* Infection Model

Virulence was assessed on representative ST59 isolates using the *C. elegans* infection model, following established techniques (Stiernagle, 2006; Wu et al., 2010). Modifications to the protocol include synchronization of the Bristol N2 *C. elegans* nematodes with bleach, allowing them to hatch and grow to the L4 stage on NGM plates inoculated with *Escherichia coli* OP50 (Stiernagle, 2006). L4, nematodes were washed from the plate with M9 buffer, washed once again in the same buffer, and approximately 30 nematodes suspended in M9 buffer added to each assay plate. *S. aureus* strains 8325 and M92 were used as positive and negative controls, respectively, and survival scored every 24 h for 5 days. Survival curves were generated using GraphPad Prism 7 (GraphPad Software, La Jolla, CA, United States). Killing rates were calibrated as [% death_Isolate_ −% death_M__92__(–ve control__)_]/[% death_8325__(+ve control)_ −% death_M__92__(–ve control__)_], with mean killing rates determined as the mean of 3–5 experimental replicates.

## Results

### ST59 Sub-Lineages Identified Based on PFGE Clusters

Pulsed field gel electrophoresis represents the traditional method for typing *S. aureus*, and for identifying different strain types. Within our complete collection of *S. aureus* strains, we identified 224 ST59 isolates, from which we generated a ST59 group specific PFGE figure (Supplementary Figure 1). As expected, the ST59 lineage was separate and distinct from our local epidemic reference strains, CMRSA 1–10, with the ST59 group sharing less than 50% similarity of DCC with the reference strains. Within the ST59 lineage, isolates could be divided into seven subgroups based on PFGE banding patterns, whereby isolates within the first subgroup (PFGE-1) shared 65% homology of DCC, isolates within PFGE groups PFGE-2, 3, 5, 6, and 7 shared 75–76% homology, and group PFGE-4 contained one isolate. Despite PFGE banding pattern similarities of isolates within a subgroup, the isolates actually showed a significant amount of variability when looking at more detailed molecular characteristics. Within each group, both methicillin-sensitive (MS-) and methicillin-resistant isolates were present, differed in *spa* types, differed in presence or absence of PVL, and had differed in the SCC*mec* types. These differences were not tied to the date or country of isolation.

### Whole Genome Sequence Analysis and Mobile Genetic Elements Associated With Our ST59

Because of the molecular heterogeneity seen within each of the PFGE groups, 47 representative isolates were selected for whole genome sequencing and a more detailed analysis. Several mobile genetic elements are proposed to be associated with evolution of the lineage, including SCC*mec*, PVL (ϕSa2), IEC, and MES, which were characterized for all the strains, with the results summarized in Table 1. An explanation of the specific IEC and MES types is shown in Table 2.

**TABLE 1:** Summary of the 47 ST59 isolates selected for whole genome sequencing.

									**IEC**							**MES**												
**WGS**	**Strain**	**MRSA/**	**Year**	**Location**	**PFGE**	***spa***	**SCC**	**PVL**	***scn***	***chp***	***sak***	***sea***	***sep***	**ϕSa3**	**IEC**	**IS1216V**	***tnp***	***tnpR***	***ermB***	***aph***	***sat***	***aadE***	**IS1216V**	***cat***	**IS1216V**	***aacA-***	**MES**	***C. elegans***
**subgroup**		**MSSA**			**group**		***mec***							**Integrase**	**Type^‡^**		**tn551**			**(3′)-III**						***aphD***	**Type^†^**	**calibrated death**
1	H509	MSSA	1993	CAN	1	172	−	−	+	+	+	+	−	−	C*	−	−	−	−	−	−	−	−	−	−	−	−	0.4017
	S2459	MSSA	2010	CAN	6	163	−	−	+	+	+	+	−	−	C*	−	−	−	−	−	−	−	−	−	−	−	−	0.2460
	H291	MRSA	1992	CAN	6	163	IV	−	+	+	+	+	−	−	C*	−	−	−	−	−	−	−	−	−	−	−	−	0.5103
	PRE	MSSA	2001	CAN	6	163	−	−	+	+	+	+	−	−	C*	−	−	−	−	−	−	−	−	−	−	−	−	0.1724
	GD321	MSSA	2010	GD	6	163	−	−	+	+	+	+	−	+	B*	−	−	−	−	−	−	−	−	−	−	−	−	0.2371
2	SSR	MSSA	2012	CAN	6	216	−	−	+	+	−	−	−	−	C	−	−	−	−	−	−	−	−	−	−	−	−	0.4333
	KAE	MSSA	2007	CAN	6	2365	−	−	+	+	−	−	−	−	C	−	−	−	−	−	−	−	−	−	−	−	−	0.1000
	290N	MSSA	2014	CAN	6	216	−	−	+	+	−	−	−	−	C	−	−	−	−	−	−	−	−	−	−	−	−	0.1517
3	GD1409	MRSA	2010	GD	3	437	IVg	−	+	−	+	−	+	+	G	+	+	+	+	+	+	−	+	+	+	−	unk-1	0.1067
	GD875	MSSA	2010	GD	3	1751	−	−	+	−	+	−	+	+	G	+	+	+	+	+	+	−	+	+	+	−	unk-1	0.1265
4	GD912	MRSA	2010	GD	4	437	IVa	−	+	+	+	−	−	+	B	+	+	+	+	+	+	+	+	−	+	−	PM9	0.0000
	GD517	MRSA	2010	GD	6	437	IVa	−	+	+	+	−	−	+	B	+	−	−	−	−	−	−	−	−	−	−	PM18	0.2111
	GD26	MRSA	2010	GD	1	437	IVa	−	+	+	+	−	−	+	B	+	−	−	−	−	−	−	−	−	−	−	PM18	0.2782
	GD38	MRSA	2010	GD	3	437	IVa	−	+	+	+	−	−	+	B	+	+	+	+	+	+	+		−		−	PM9	0.1522
	GD31	MRSA	2010	GD	3	441	IVa	−	+	+	+	−	−	+	B	+	+	+	+	+	+	+	+	−	+	−	PM9	0.0000
	GD3.1	MRSA	2010	GD	5	441	IVa	−	+	+	+	−	−	+	B	+	+	+	+	+	+	+	+	−	+	−	PM9	0.1211
	GD1633	MRSA	2010	GD	2	441	IVa	+	+	+	+	−	−	−	C**	+	+	+	+	+	+	+	+	−	+	−	PM9	0.0278
	GD75	MRSA	2010	GD	3	437	IVa	−	+	+	+	−	−	+	B	+	+	+	+	+	+	+	+	−	+	−	PM9	0.0692
	GD19	MRSA	2010	GD	5	437	IVa	−	+	+	+	−	−	+	B	+	+	+	+	+	+	+	+	−	+	−	PM9	0.1703
	GD81	MRSA	2010	GD	3	437	IVa	−	+	+	+	−	−	+	B	+	+	+	+	+	+	+	+	−	+	−	PM9	0.0775
	GD15	MRSA	2010	GD	3	437	IVa	−	+	+	+	−	−	+	B	+	+	+	+	+	+	+	+	−	+	−	PM9	0.1827
	GD1907	MRSA	2010	GD	3	437	IVa	−	+	+	+	−	−	+	B	+	+	+	+	+	+	+	+	−	+	−	PM9	0.0324
	GD1958	MRSA	2010	GD	1	437	IVa	−	+	+	+	−	−	+	B	+	+	+	+	+	+	+	+	−	+	−	PM9	0.1479
5	SA28	MSSA	2003	CAN	2	437	−	+	+	+	−	−	−	−	C	+	+	+	+	+	+	+	+	−	+	−	PM9	0.2010
	GD52	MRSA	2010	GD	2	437	IVa	+	+	+	−	−	−	−	C	+	+	+	+	+	+	+	+	−	+	−	PM9	0.0702
	GD858	MRSA	2010	GD	2	4347	IVa	+	+	+	−	−	−	−	C	+	+	+	+	+	+	+	+	−	+	−	PM9	0.3086
	GD1054	MRSA	2010	GD	5	437	IVa	+	+	+	+	+	−	−	C*	+	+	+	+	+	+	+	+	−	+	−	PM9	0.1360
	GD27	MRSA	2010	GD	4	441	IVa	+	+	+	+	+	−	−	C*	+	+	+	+	+	+	+	+	−	+	−	PM9	0.2447
	GD1605	MSSA	2010	GD	1	1751	−	−	+	+	−	−	−	−	C	+	+	+	+	+	+	+	+	+	+	−	PM1	0.3899
	GD251	MSSA	2010	GD	5	3485	−	−	+	+	−	−	−	−	C	+	+	+	+	+	+	+	+	+	+	−	PM1	0.0604
	GD1171	MSSA	2010	GD	5	8886	−	−	+	+	−	−	−	−	C	+	+	+	+	+	+	+	+	+	+	−	PM1	0.5861
	GD843	MSSA	2010	GD	1	8886	−	−	+	+	−	−	−	−	C	+	−	−	−	−	−	−	−	−	−	−	PM18	0.2307
	GD1941	MRSA	2010	GD	5	437	IVa	−	+	+	−	−	−	−	C	+	+	+	+	+	+	+	+	+	+	−	PM1	0.1778
	GD910	MSSA	2010	GD	5	4134	−	−	+	+	−	−	−	−	C	+	−	−	−	−	−	−	−	−	−	−	PM18	0.0000
	GD1976	MRSA	2010	GD	5	437	IV	−	+	+	−	−	−	−	C	+	+	+	+	+	+	+		−		−	PM9	0.0333
	GD511	MSSA	2010	GD	2	437	−	+	+	+	−	−	−	−	C	+	+	+	+	+	+	+	+	−	+	−	PM9	0.2046
									**IEC**							**MES**												
	GD1176	MSSA	2010	GD	7	437	−	−	+	+	−	−	−	−	C	+	−	−	−	−	−	−	−	−	−	−	PM18	0.0707
	GD1038	MRSA	2010	GD	3	437	IVg	−	+	+	−	−	−	−	C	+	+	+	+	+	+	+	+	+	+	−	PM1	0.1000
	GD954	MSSA	2010	GD	7	437	−	−	+	+	−	−	−	−	C	+	+	+	+	+	+	+	+	−		−	PM9	0.0679
6	MS07MS-625	MRSA	2007	CAN	2	437	Vb	+	+	+	−	−	−	−	C	+	+	+	+	+	+	+	+	−	+	−	PM9	0.2371
	CAN20	MRSA		MAL	2	3590	Vb	+	+	+	−	−	−	−	C	+	+	+	+	+	+	+	+	−	+	−	PM9	0.0693
	C11224	MRSA	2006	CAN	2	437	Vb	+	+	+	−	−	−	−	C	+	+	+	+	+	+	+	+	+	+	−	PM1	0.0636
	C3714	MRSA	2006	CAN	2	unk	Vb	+	+	+	−	−	−	−	C	+	+	+	+	+	+	+	+	+	+	−	PM1	0.4278
	GD21	MRSA	2010	GD	2	437	Vb	+	+	+	−	−	−	−	C	+	+	+	+	+	+	+	+	+	+	−	PM1	0.0000
	GD1068	MRSA	2010	GD	7	437	Vb	−	+	+	−	−	−	−	C	+	+	+	+	+	+	+	+	+	+	−	PM1	0.1269
	GD1199	MRSA	2010	GD	2	437	Vb	+	+	+	−	−	−	−	C	+	+	+	+	+	+	+	+	+	+	−	PM1	0.1037
	C889b	MRSA	2008	CAN	2	437	Vb	+	+	+	−	−	−	−	C	+	+	+	+	+	+	+	+	+	+	−	PM1	0.2376

Country and year of isolation are indicated, as well as relevant molecular characteristics and their associated virulence, as measured in the *C. elegans* infection model. MRSA, methicillin resistant *Staphylococcus aureus*; MSSA, methicillin sensitive *Staphylococcus aureus*; PFGE, Pulsed field gel electrophoresis; *spa*, staphylococcal protein A; SCC*mec*, Staphylococcal cassette chromosome *mec*; PVL, Panton-Valentine leucocidin; IEC, immune evasion cluster; *scn*, complement inhibitor; *chp*, chemotaxis-inhibiting protein; *sak*, Staphylokinase precursor; *sea*, staphylococcal enterotoxin A precursor; *sep*, staphylococcal enterotoxin P precursor; MES, mobile element structure; *tnp* tn551, tn551 transposase; *tnpR*, resolvase; *ermB*, erythromycin resistance transferase; *aph(3′)-III*, aminoglycoside 3′-phosphotransferase; *sat*, streptothricin acetyltransferase; *aadE*, streptomycin adenylyltransferase; *cat*, chloramphenicol acetyltransferase; *aacA-aphD*, bifunctional aminoglycoside modifying enzyme; ^‡^, as defined by Hung et al. (2016) and this study; ^†^, as defined by Hung et al. (2012) and this study; +, positive for trait/gene; −, negative for trait/gene; * and **, IEC variants as defined by Table 2 of this study; unk, unknown.

**TABLE 2:** Immune Evasion Complex (IEC) and Mobile Elements Structure (MES) types, along with their associated genes, detected in the ST59 isolates.

**IEC type**^‡^	***scn***	***chp***	***sak***	***sea***	***sep***	**ϕSa3 integrase**	**MES type**^†^	**IS1216V**	***tnp*_tn551**	***tnpR***	***ermB***	***aph(3′)-III***	***sat***	***aadE***	***cat***	***aacA-aphD***
C*	+	+	+	+	−	−	PM1	+	+	+	+	+	+	+	+	−
C**	+	+	+	−	−	−	PM9	+	+	+	+	+	+	+	−	−
C	+	+	−	−	−	−	PM18	+	−	−	−	−	−	−	−	−
B*	+	+	+	+	−	+	6272-2	+	+	+	+	+	−	−	+	+
B**	+	+	+	−	+	+	unk-1	+	+	+	+	+	+	−	+	−
B	+	+	+	−	−	+	unk-2	+	+	+	+	−	−	−	+	−
G	+	−	+	−	+	+	unk-3	+	+	+	+	−	−	−	+	+
G**	+	−	+	−	−	+	unk-4	−	+	−	+	+	−	−	+	+
X	+	−	−	−	−	−	unk-5	+	−	−	+	+	−	−	−	+
unk-1	−	+	−	−	−	−	unk-6	−	+	−	+	−	−	−	+	−
							unk-7	+	+	+	+	−	−	−	−	−
							unk-8	+	+	+	+	−	−	−	−	+
							unk-9	−	+	+	+	+	+	+	+	+

scn, complement inhibitor; *chp*, chemotaxis-inhibiting protein; *sak*, Staphylokinase precursor; *sea*, staphylococcal enterotoxin A precursor; *sep*, staphylococcal enterotoxin P precursor; tnp tn551, tn551 transposase; *tnpR*, resolvase; *ermB*, erythromycin resistance transferase; *aph(3′)-III*, aminoglycoside 3′-phosphotransferase; *sat*, streptothricin acetyltransferase; *aadE*, streptomycin adenylyltransferase; *cat*, chloramphenicol acetyltransferase; *aacA-aphD*, bifunctional aminoglycoside modifying enzyme; ^‡^, as defined by Hung et al. (2016) and this study; ^†^, as defined by Hung et al. (2012) and this study; * and **, IEC variants; +, positive for gene; −, negative for gene; unk, unknown.

### ST59 Evolution

**FIGURE 1:**
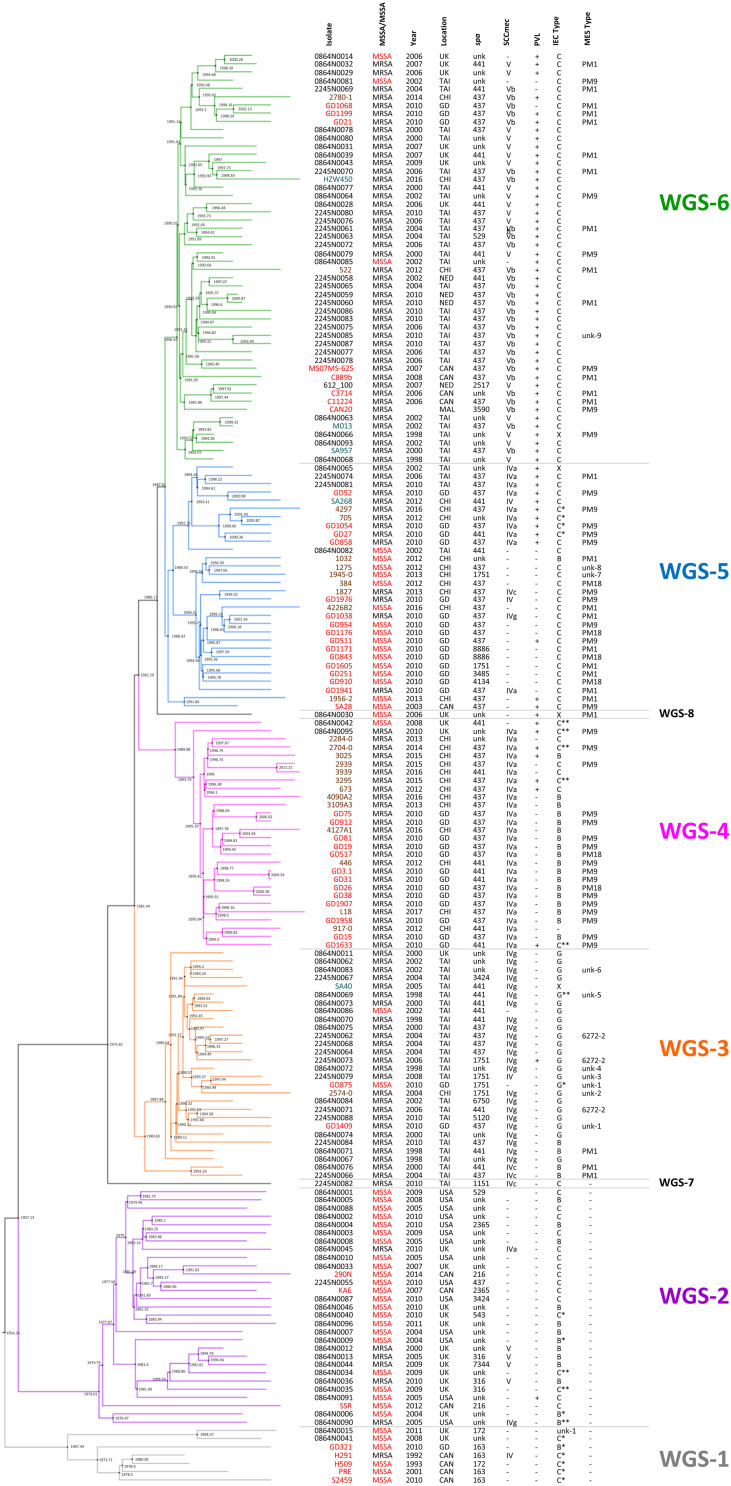
Maximum clade credibility tree showing the phylogenetic relationship between our ST59 isolates, along with global isolates from studies by Ward et al. (2016) and Pang et al. (2020). Branch colors represent the 6 sub-lineages, with WGS-1 represented by gray, WGS-2 by purple, WGS-3 by orange, WGS-4 by pink, WGS-5 by blue and WGS-6 by green. Nodes are marked with black circles, with the estimated node date indicated. Isolates from our study are indicated with red font, isolates from Ward et al. by black font, isolates from Pang et al. by brown font, and sequences downloaded from NCBI in teal font. Methicillin resistance (with MSSA indicated with red font), year and country of isolation, *spa* type, SCC*mec* type, PVL carriage, IEC type and MES type are indicated for each strain. MRSA, methicillin resistant *Staphylococcus aureus*; MSSA, methicillin sensitive *Staphylococcus aureus*; *spa*, staphylococcal protein A; SCC*mec*, Staphylococcal cassette chromosome *mec*; PVL, Panton-Valentine leucocidin; IEC, immune evasion cluster; MES, mobile element structure; CAN, Canada; CHI, China; GD, Guangdong, China; MAL, Malaysia; NED, Netherlands; TAI, Thailand; UK, United Kingdom; USA, United States of America; unk, unknown; +, trait is present; –, trait is absent.

Previous studies have suggested that ST59 evolved concurrently in both North America (NA), previously referred to as the United States lineage and East Asia (EA), with strains from each region belonging to separate and distinct sub-lineages (Ward et al., 2016), with mobile genetic elements playing a part (Hung et al., 2016). For a more detailed look into the evolution of the ST59 lineage, a Bayesian inference phylogenomic tree, comprised of both the monomorphic SNPs and insertions/deletions identified by snippy, was generated incorporating our isolates (red font), as well as select isolates from the Ward et al. (2016) (blue font) and Pang et al. (2020) (green font) studies, and ST59 sequences downloaded from NCBI (black font). Molecular typing data was determined for the downloaded sequences, with IEC type identified for all isolates, and MES type for a random selection of isolates. The phylogenetic tree, along with the associated molecular information, is shown in Figure 1, with detailed strain data provided in Supplementary Table 1. Analysis of the tree identified separate sub-lineages, WGS-1 to 6. The NA (WGS-2) and EA (WGS-3 to 6) sub-lineages are seen evolving concurrently around 1957 (95% HPD interval = [1955, 1967]), with the isolates in sub-lineage WGS-1 appearing to diverge earlier than the previously described NA/EA split, branching off from the others in 1954 (95% HPD interval = [1946, 1964]). An interesting finding was the observation that all isolates in the NA sub-lineages (WGS-1 and WGS-2) lacked an MES element, while isolates in the EA sub-lineages (WGS-3 to 6) contained an MES element. The various sub-lineages were further defined by their SCC*mec* type, PVL carriage, and IEC type, with MES type being more random (summarized in Figure 1 and Supplementary Table 1, with IEC and MES types as defined in Table 2). Two isolates, 2245N0082 and 0864N0030 did not fall into any of these sub-groups, each standing alone as a distinct branch labeled as WGS-7 and WGS-8, respectively.

### Methicillin Resistance in the ST59 SubLineages

As seen in Figure 1, sub-lineages WGS-1, 2 and a branch of WGS-5 were comprised primarily of MSSA isolates, while WGS-3, 4, 6, and a branch of WGS-5 were comprised primarily of MRSA isolates. Within each sub-group, however, both MRSA and MSSA were identified. *In silico* genome to genome distance calculations were done on the most closely positioned MRSA/MSSA pairs to demonstrate their high degree of relatedness, with the results summarized in Table 3. Similarities within groups ranged from 99.5 to 99.8%, while comparisons between groups produced similarities ranging from 98.1 to 99.1%.

**TABLE 3:** *In silico* genome to genome distance calculations between the most closely related MRSA and MSSA pairs in each sub-lineage, as well as between the most directly descended isolates in each group.

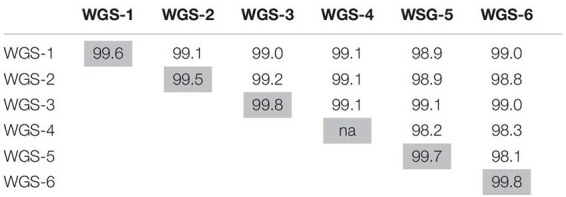

*Light gray shading denotes comparisons between the most closely positioned MRSA/MSSA pairs within each sub-lineage: H291/H509 (WGS-1); 0864N0045/0864N0003 (WGS-2); 2245N0079/GD875 (WGS-3); there was not a closely related pair for WGS-4; GD1038/GD954 (WGS-5); 0864N0032/0864N0014 (WGS-6). For comparisons between sub-groups, the following stains were used: 0864N0015 (WGS-1), 0864N0006 (WGS-2), 0864N0076 (WGS-3), 0864N0042 (WGS-4), 1956-2 (WGS-5), and 0864N0068 (WGS-6).*

### ST59 Virulence Assessed in the *C. elegans* Infection Model

Having defined the distinct ST59 sub-lineages and determining that they each have relatively homogenous molecular characteristics, we sought to determine if there were any differences in virulence between the groups. All 47 of our isolates, with multiple representatives from each of the 6 sub-lineages, had virulence assessed using the *C. elegans* infection model, with the results presented in Table 1. In general, ST59 virulence was found to be low, with calibrated killing rates ranging from 0.00 to 0.59, although the majority of isolates (39/47) were below 0.25. While subgroup WGS-1 had a greater number of members with relatively higher virulence, there did not appear to be any distinct pattern of virulence associated with the other sub-lineages. Calibrated deaths for WGS-1 ranged from 0.17 to 0.51, but as mentioned, 4 of the 5 isolates in this group were near or above 0.25. Calibrated deaths for WGS-2 ranged from 0.1 to 0.43, and both isolates in WGS-3 had calibrated deaths of approximately 0.1. Calibrated deaths for WGS-4 ranged from 0.00 to 0.28, for WGS-5 from 0.00 to 0.59, and for WGS-6 ranged from 0.00 to 0. 43. In general, the molecular characteristics, including *spa* type, SCC*mec* type, PVL presence an IEC type, didn’t appear to correlate with *C. elegans* toxicity. While strains in WGS-1 with higher toxicity carried the *sea* gene, other strains with higher relative toxicity (such as GD1171) did not carry the gene, and PRE carried it but had low toxicity.

## Discussion

The origins and evolution of *S. aureus* CC59 has received a significant amount of interest in recent years due to its high prevalence in Asian countries. Previous studies have attempted to explain evolution of the lineage, not only identifying separate sub-lineages, but also identifying factors that contributed to expansion and success of the each of the sub-lineage. Studies by Hung et al. (2016) and Pang et al. (2020) focused on ST59 success on a local scale, investigating the lineage in Taiwan and mainland China, respectively, while a study by Ward et al. (2016) examined evolution of the lineage on the global scale. All studies noted that acquisition of mobile genetic elements such as IEC, MES, SCC*mec*, and PVL contributed to diversification of the lineage. However, an important finding made by Ward et al. was the identification of two major clades of *S. aureus* ST59 (one from the North America and a second from East Asia), which were calculated to have evolved separately but concurrently, with no direct exchange between them. This dispelled the notion that the EA clade descended from the NA one, which had previously been suggested based on the earlier reporting of USA1000 (DeLeo et al., 2010). To expand our knowledge of this important lineage we used whole genome sequencing to analyze our ST59 isolates, with the goal of determining how they fit into the previously proposed CC59 evolution description.

**FIGURE 2:**
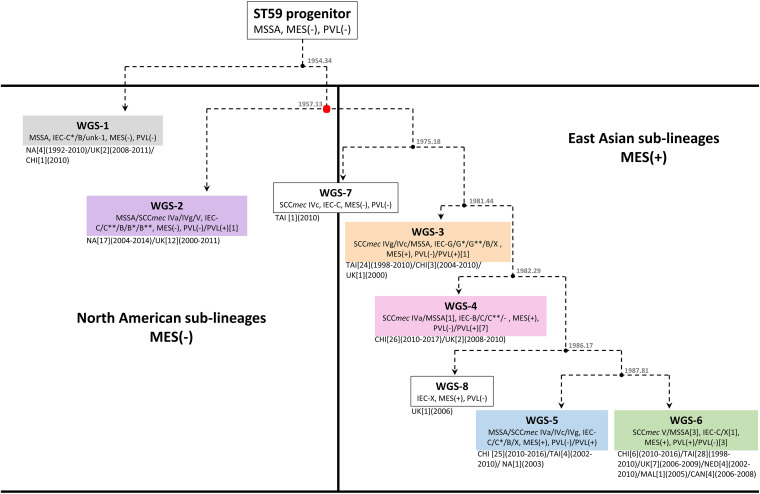
Proposed evolutionary history of *S. aureus* ST59 showing the separate North American and East Asian sub-lineages. Box colors correspond to the sub-group (WGS-1 to 6) colors described in Figure 1. Nodes are represented by black circles with the estimated node date (from BEAST analysis) shown. Molecular data is indicated for each branch, with the numbers of isolates carrying a trait indicated in square brackets when it represents an exception. Location of isolation is shown, along with the number of isolates from each location in square brackets, and the years of isolation in round brackets. The red node represents the point at which the NA and EA sub-lineages co-evolved, as described by Ward et al. (2016).

Bayesian inference phylogenomic (BEAST) analysis of our 47 isolates, combined with previously described representative global isolates, allowed us to prepare an updated evolutionary hypothesis for the lineage, which is presented in Figure 2. Similar to the model proposed by Hung et al., our model predicts the ST59 progenitor to be MSSA, PVL(-), and MES(-) (Hung et al., 2016). Likewise, similar to the model presented by Ward et al. (2016), our model proposes that distinct NA (WGS-2) and EA (WGS-3 to 6) clades co-evolved at around the same time, in 1957 (red node), a date which corresponded well with their reported date for co-evolution in the late 1950s to mid 1960s. Within the NA sub-lineage there was minimal diversification or evolution of ST59. In contrast, the EA sub-lineages were more numerous, with 4 large sub-groups identified, each of which showed significant expansion and evolution. Our results are in keeping with previous studies that have identified a large amount of diversity within the EA lineage. Chen et al. (2013) identified both a virulent Taiwan clone and a commensal Asian-Pacific clone, while Hung et al. (2016) noted that CC59 in Taiwan acquire divergent mobile genetic elements, and followed 2 evolutionary branches with subsequent diversification.

A recent study by Pang et al. (2020) also noted that the ST59 underwent significant diversification and geographic expansion in mainland China during the 1980s and 1990s. When comparing our EA sub-groups to previously described groups, we see that WGS-4 is representative of the Asia Pacific clone [MRSA IV/IEC-B/PVL(−)] (Chen et al., 2013), and WGS-3 represents one of the two major branch in Taiwan [MRSA IVg/IEC-G/PVL(−)] (Hung et al., 2016). Similarly, WGS-6 is representative of the other major branch in Taiwan [MRSA V_T_/IEC-C/PVL(+)], as is WGS-5 [MRSA IVa/IEC-C/PVL(+) or MSSA/IEC-C/PVL(−)] (Hung et al., 2016). Where our results differed from previous studies is that we identified another distinct sub-lineage (WGS-1), which diverged earlier to the previously described NA and EA ones, calculated to have evolved in 1954, prior to the reported NA/EA split. Similar to the WGS-2 NA isolates, WGS-1 isolates were all MES(-), and represent some of the earliest collection dates (1992 and 1993) reported for ST59. Interestingly, the continued isolation of WGS-1 (as recently as 2011), and its presence in regions such as the United Kingdom and mainland China, shows that it still persists in small numbers, and suggests that there was early intercontinental transfer of this sub-lineage. It does not, however, appear to have gained dominance in any region, possibly due to antibiotic selective pressures.

As mentioned, both WGS-1 and WGS-2, which constitute the NA lineages, lack a MES element and the antibiotic resistance genes contained therein, which is in stark contrast to isolates in the East Asian sub-lineages which all contain an MES element. In fact, MES appears to be the major distinguishing feature that separates these 2 continental lineages, as the other traits such as IEC, SCC*mec* and PVL are shared by isolates in both groups. The presence of MES in EA lineages has been attributed to the high levels of non-prescription antibiotic use seen in many Asian countries (MorganI, Okeke et al., 2011) which has created selective pressures favoring the presence of resistance cassettes, such as MES, in strains that have become epidemic there. On the other hand, the fitness costs associated with unnecessary resistance cassettes in regions with low antibiotic use (such as NA) likely favor MES(-) strains (Anderson et al., 2014; Melnyk et al., 2015; Ward et al., 2016). Within the EA sub-lineages, the specific MES type and associated resistance genes present are likely influenced by regional antibiotic use and specific selection pressures, as a significant amount of variability was noted within each sub-group. An interesting observation is the existence of a single MES(-) MRSA (WGS-7) isolated in Taiwan in 2010. BEAST analysis predicted that this strain appeared prior to diversification of the EA sub-lineages, in approximately 1975. This isolate hints at the possibility of transfer of a NA like isolate to EA, which then acquired the MES element, providing the necessary antibiotic resistance for subsequent dispersal and dominance of ST59 in EA. The strain continues to exist in small numbers in the region, but never attained dominance in EA due to the lack of MES.

A deeper look at factors that may be responsible for the dominance of ST59 in EA has focused on mobile genetic elements such as IEC, MES, SCC*mec*, and PVL. Others have postulated that these elements were the primary factors that drove evolution and allowed CC59 to become such a successful lineage in EA (Hung et al., 2016; Pang et al., 2020). Our results, however, showed enough diversity within each sub-group for most of the traits to make it unclear if they are actually responsible for driving appearance and success of the sub-lineages, or if they are merely being gained, lost or modified based on local selection conditions. IEC is interesting because the earliest evolved lineage (WGS-1) carries IEC-C^∗^, which contains the greatest number of virulence genes (*scn*, *chp*, *sak*, and *sea*). However, as the ST59 lineage evolved the complexes appeared to contain progressively fewer of these genes. It has been suggested that IEC associated genes on ϕSa3 are associated with strain virulence (McClure et al., 2018; Kashif et al., 2019), which could play a role in the ability of a strain to become dominant in a region. The Taiwan clone (MRSAV_T_-PVL(+); WGS-6) is documented to cause more severe infections (Chen et al., 2005; Huang et al., 2007), representing 73% of infections in Taiwanese children (Huang et al., 2008), and 71.1% of clinical MRSA isolates (Chen et al., 2005). It is, however, one of the latest sub-groups to appear in the evolutionary pathway and contains IEC-C with only 2 of the genes (*scn* and *chp*), suggesting that its success is unrelated to the complexity of IEC. PVL may in fact be more tied to the virulence of the Taiwan sub-lineage, as was previously suggested (Chen et al., 2005), as most isolates in sub-group WGS-6 are PVL(+). However, the presence of PVL in all of the other sub-lineages, including the colonizing Asian-Pacific clone (ST59-MRSAIV-PVL(-); WGS-4), as well as its absence in some isolates from WGS-6, calls into question its driving role in the success of the sub-group. The role of SCC*mec* in the context of ST59 evolution is also unclear. Hung et al. (2016) noted that SCC*mec* V between Taiwan and mainland China strains differed, as did SCC*mec* IV between the 2 regions. They also observed that the separate ST59 clades in Taiwan independently acquired distinct SCC*mec* types (IVg and V_T_), concluding that the acquisition of specific SCC*mec* types helped the clades achieve dominance in their local regions.

Our results indicated that while certain SCC*mec* types (or lack of one) typified many of the sub-lineages, in each group where both MSSA and MRSA were seen, and often multiple SCC*mec* types were encountered. In fact, both MRSA and MSSA were identified as coexisting amongst the earliest reported isolates (1992 and 1993, respectively), in the earliest sub-lineage to have evolved (WGS-1). Genome to Genome distance calculation between MRSA and MSSA pairs within each sub-group indicated that they were closely related to each other, suggesting that they share the same genetic background and that SCC*mec* is readily being gained/lost from the strain. As such, our data suggests that while mobile genetic elements such as IEC, PVL, and SCC*mec* have types commonly associated with each of the sub-lineages, the traits cannot be used as rigid definers of the lineages, as was suggested by Hung et al. (2016). Rather, the high degree of diversity for each of these traits within a sub-lineage suggests their presence is related to local selective pressures, as was suggested by Pang et al. (2020) when they noted the significant diversification of the lineage in mainland China.

Having identified the various ST59 sub-lineages and identified some of the virulence associated elements that they contain, we sought to determine if there were any differences between them in terms of toxicity in the *C. elegans* infection model. We were surprised to note that there was very little difference in toxicity between the ST59 sub-groups, despite the differences in IEC related virulence gene carriage. Overall, we found that the lineage had a low level of *C. elegans* toxicity, particularly when compared to other lineages such as ST398 (including isolates from the same region in Guangdong) (Kashif et al., 2019), USA300 (ST8), USA400 (ST1) (Wu et al., 2010; Obasuyi et al., 2020), ST30 and ST152 (Obasuyi et al., 2020). No discernible link was noted between toxicity and virulence gene carriage in most sub lineages, although there were more isolates in WGS-1 with a slightly higher level of toxicity. This group carries IEC-C^∗^, which contains the greatest number of virulence genes, including *scn*, *chp*, *sak*, and *sea*, all of which have been implicated in *S. aureus* virulence (de Haas et al., 2004; Bokarewa et al., 2006; Rooijakkers et al., 2007; Ortega et al., 2010). IEC is, however, unlikely to be the main contributor to virulence as strains such as GD1171 and GD1605 which had similarly elevated levels of toxicity, but carried IEC-C with only *scn* and *chp*. Conversely, PRE carried IEC-C^∗^, but had a very low level of *C. elegans* toxicity. Other variations must therefore exist in the genomes of these sub-lineages which contribute to virulence.

## Conclusion

Our study was able to construct an updated evolutionary story for the ST59 lineage. Our model not only included the co-evolution of North American and East Asian sub-lineages previously described in other studies, but also identified and older North American sub-lineage that was calculated to have emerged prior to the previously described NA/EA groups. We identified MES as being the major difference between NA an EA lineages, with NA sub-lineages showing minor expansion, while EA lineages showed significant diversification. Mobile genetic elements, such as IEC, PVL, and SCC*mec*, showed significant variability within each sub-group and likely represent local selective pressures, rather than major defining characteristic for each group. And finally, while our data cannot directly show that ST59 evolved in NA and was then transported to EA, the fact that the earliest appearing sub-group had NA characteristics [MES (-)] and was predominantly isolated in NA with some of the earliest recorded ST59 isolates, combined with the presence of a MES(-) isolate in Taiwan, predicted to have appeared prior to diversification of the EA lineages, hints that this may be the case. Further studies will of course be needed to confirm this supposition.

## Data Availability Statement

The datasets presented in this study can be found in online repositories. The names of the repository/repositories and accession number(s) can be found in the article/Supplementary Material.

## Ethics Statement

The protocols were approved by the University of Calgary Conjoint Health Research Ethics under the Certification No: REB13-0219, and the Ethics Committee of the First Affiliated Hospital/School of Clinical Medicine of Guangdong Pharmaceutical University under the Ethics No. 2011(1), respectively. Written informed consent was obtained from all participants.

## Author Contributions

KZ conceived, designed, and supervised the work. J-AM, SL, AN, GD, and OO performed the experiments and analyzed data. PG constructed Bayesian inference phylogenomic tree. SC and JC collected and provided the clinical isolates. J-AM and KZ structured and drafted the manuscript. JC and KZ edited the manuscript. All authors reviewed and approved the manuscript.

## Conflict of Interest

The authors declare that the research was conducted in the absence of any commercial or financial relationships that could be construed as a potential conflict of interest.

## Footnotes

1 https://github.com/tseemann/snippy
