# Supplementary material for: Staphylococcus aureus ST59: Concurrent but Separate Evolution of North American and East Asian Lineages
Source: Front Microbiol. 2021 Feb 10;12:631845. doi: 10.3389/fmicb.2021.631845 (PMC7902796; doi:10.3389/fmicb.2021.631845)
Supplement: Supplementary file 1 [file Table_1.DOCX]

**Supplementary Table 1.** Molecular characteristics of all ST59 isolates used in the Bayesian inference phylogenomic analysis.

|  |  |  |  |  |  |  |  | **IEC** | | | | | | |  | | **MES** | | | | | | | | | | | | |
| --- | --- | --- | --- | --- | --- | --- | --- | --- | --- | --- | --- | --- | --- | --- | --- | --- | --- | --- | --- | --- | --- | --- | --- | --- | --- | --- | --- | --- | --- |
| **WGS subgroup** | **Strain** | **MRSA/MSSA** | **Year** | **Location** | ***spa*** | **SCC*mec*** | **PVL** | ***scn*** | ***chp*** | ***sak*** | ***sea*** | ***sep*** | **φSa3 Integrase** | **IEC Type**^‡^ | |  | | **IS1216V** | ***tnp* tn551** | ***tnpR*** | ***ermB*** | ***aph(3')-III*** | ***sat*** | ***aadE*** | **IS1216V** | ***cat*** | **IS1216V** | ***aacA-aphD*** | **MES Type**^†^ |
| **WGS-5** | **0864N0014** | MSSA | 2006 | UK | unk | - | + | + | + | - | - | - | - | C | |  | |  |  |  |  |  |  |  |  |  |  |  |  |
|  | **0864N0032** | MRSA | 2007 | UK | 441 | V | + | + | + | - | - | - | - | C | |  | | + | + | + | + | + | + | + |  | + |  | - | PM1 |
|  | **0864N0029** | MRSA | 2006 | UK | unk | V | + | + | + | - | - | - | - | C | |  | |  |  |  |  |  |  |  |  |  |  |  |  |
|  | **0864N0081** | MSSA | 2002 | TAI | unk | - | - | + | + | - | - | - | - | C | |  | |  | + | + | + | + | + | + |  | - |  | - | PM9 |
|  | **2245N0069** | MRSA | 2004 | TAI | 441 | Vb | - | + | + | - | - | - | - | C | |  | | + | + | + | + | + | + | + | + | + | + | - | PM1 |
|  | **2780-1** | MRSA | 2014 | CHI | 437 | Vb | + | + | + | - | - | - | - | C | |  | |  |  |  |  |  |  |  |  |  |  |  |  |
|  | **GD1068** | MRSA | 2010 | GD | 437 | Vb | - | + | + | - | - | - | - | C | |  | | + | + | + | + | + | + | + | + | + | + | - | PM1 |
|  | **GD1199** | MRSA | 2010 | GD | 437 | Vb | + | + | + | - | - | - | - | C | |  | | + | + | + | + | + | + | + | + | + | + | - | PM1 |
|  | **GD21** | MRSA | 2010 | GD | 437 | Vb | + | + | + | - | - | - | - | C | |  | | + | + | + | + | + | + | + | + | + | + | - | PM1 |
|  | **0864N0078** | MRSA | 2000 | TAI | 437 | V | + | + | + | - | - | - | - | C | |  | |  |  |  |  |  |  |  |  |  |  |  |  |
|  | **0864N0080** | MRSA | 2000 | TAI | unk | V | + | + | + | - | - | - | - | C | |  | |  |  |  |  |  |  |  |  |  |  |  |  |
|  | **0864N0031** | MRSA | 2007 | UK | unk | V | + | + | + | - | - | - | - | C | |  | |  |  |  |  |  |  |  |  |  |  |  |  |
|  | **0864N0039** | MRSA | 2007 | UK | 441 | V | + | + | + | - | - | - | - | C | |  | |  | + | + | + | + | + | + |  | + | + | - | PM1 |
|  | **0864N0043** | MRSA | 2009 | UK | unk | V | + | + | + | - | - | - | - | C | |  | |  |  |  |  |  |  |  |  |  |  |  |  |
|  | **2245N0070** | MRSA | 2006 | TAI | 437 | Vb | + | + | + | - | - | - | - | C | |  | | + | + | + | + | + | + | + | + | + | + | - | PM1 |
|  | **HZW450** | MRSA | 2016 | CHI | 437 | Vb | + | + | + | - | - | - | - | C | |  | |  |  |  |  |  |  |  |  |  |  |  |  |
|  | **0864N0077** | MRSA | 2000 | TAI | 441 | V | + | + | + | - | - | - | - | C | |  | |  |  |  |  |  |  |  |  |  |  |  |  |
|  | **0864N0064** | MRSA | 2002 | TAI | unk | V | + | + | + | - | - | - | - | C | |  | |  | + | + | + | + | + | + |  | + |  |  | PM9 |
|  | **0864N0028** | MRSA | 2006 | UK | 441 | V | + | + | + | - | - | - | - | C | |  | |  |  |  |  |  |  |  |  |  |  |  |  |
|  | **2245N0080** | MRSA | 2010 | TAI | 437 | V | + | + | + | - | - | - | - | C | |  | |  |  |  |  |  |  |  |  |  |  |  |  |
|  | **2245N0076** | MRSA | 2006 | TAI | 437 | Vb | + | + | + | - | - | - | - | C | |  | |  |  |  |  |  |  |  |  |  |  |  |  |
|  | **2245N0061** | MRSA | 2004 | TAI | 437 | Vb | + | + | + | - | - | - | - | C | |  | |  | + | + | + | + | + | + | + | + |  | - | PM1 |
|  | **2245N0063** | MRSA | 2004 | TAI | 529 | Vb | + | + | + | - | - | - | - | C | |  | |  |  |  |  |  |  |  |  |  |  |  |  |
|  | **2245N0072** | MRSA | 2006 | TAI | 437 | Vb | + | + | + | - | - | - | - | C | |  | |  |  |  |  |  |  |  |  |  |  |  |  |
|  | **0864N0079** | MRSA | 2000 | TAI | 441 | V | + | + | + | - | - | - | - | C | |  | | + | + | + | + | + | + | + |  | - |  | - | PM9 |
|  | **0864N0085** | MSSA | 2002 | TAI | unk | - | + | + | + | - | - | - | - | C | |  | |  |  |  |  |  |  |  |  |  |  |  |  |
|  | **522** | MRSA | 2012 | CHI | 437 | Vb | + | + | + | - | - | - | - | C | |  | | + | + | + | + | + | + | + | + | + | + | - | PM1 |
|  | **2245N0058** | MRSA | 2002 | Ned | 441 | Vb | + | + | + | - | - | - | - | C | |  | |  |  |  |  |  |  |  |  |  |  |  |  |
|  | **2245N0065** | MRSA | 2004 | TAI | 437 | Vb | + | + | + | - | - | - | - | C | |  | |  |  |  |  |  |  |  |  |  |  |  |  |
|  | **2245N0059** | MRSA | 2010 | NED | 437 | Vb | + | + | + | - | - | - | - | C | |  | |  |  |  |  |  |  |  |  |  |  |  |  |
|  | **2245N0060** | MRSA | 2010 | NED | 437 | Vb | + | + | + | - | - | - | - | C | |  | |  | + | + | + | + | + | + | + | + |  | - | PM1 |
|  | **2245N0086** | MRSA | 2010 | TAI | 437 | Vb | + | + | + | - | - | - | - | C | |  | |  |  |  |  |  |  |  |  |  |  |  |  |
|  | **2245N0083** | MRSA | 2010 | TAI | 437 | Vb | + | + | + | - | - | - | - | C | |  | |  |  |  |  |  |  |  |  |  |  |  |  |
|  | **2245N0075** | MRSA | 2006 | TAI | 437 | Vb | + | + | + | - | - | - | - | C | |  | |  |  |  |  |  |  |  |  |  |  |  |  |
|  | **2245N0085** | MRSA | 2010 | TAI | 437 | Vb | + | + | + | - | - | - | - | C | |  | |  | + | + | + | + | + | + |  | + |  | + | unk-9 |
|  | **2245N0087** | MRSA | 2010 | TAI | 437 | Vb | + | + | + | - | - | - | - | C | |  | |  |  |  |  |  |  |  |  |  |  |  |  |
|  | **2245N0077** | MRSA | 2006 | TAI | 437 | Vb | + | + | + | - | - | - | - | C | |  | |  |  |  |  |  |  |  |  |  |  |  |  |
|  | **2245N0078** | MRSA | 2006 | TAI | 437 | Vb | + | + | + | - | - | - | - | C | |  | |  |  |  |  |  |  |  |  |  |  |  |  |
|  | **MS07MS-625** | MRSA | 2007 | CAN | 437 | Vb | + | + | + | - | - | - | - | C | |  | | + | + | + | + | + | + | + | + | - | + | - | PM9 |
|  | **C889b** | MRSA | 2008 | CAN | 437 | Vb | + | + | + | - | - | - | - | C | |  | | + | + | + | + | + | + | + | + | + | + | - | PM1 |
|  | **612_100** | MRSA | 2007 | NED | 2517 | V | + | + | + | - | - | - | - | C | |  | |  |  |  |  |  |  |  |  |  |  |  |  |
|  | **C3714** | MRSA | 2006 | CAN | unk | Vb | + | + | + | - | - | - | - | C | |  | | + | + | + | + | + | + | + | + | + | + | - | PM1 |
|  | **C11224** | MRSA | 2006 | CAN | 437 | Vb | + | + | + | - | - | - | - | C | |  | | + | + | + | + | + | + | + | + | + | + | - | PM1 |
|  | **CAN20** | MRSA |  | MAL | 3590 | Vb | + | + | + | - | - | - | - | C | |  | | + | + | + | + | + | + | + | + | - | + | - | PM9 |
|  | **0864N0063** | MRSA | 2002 | TAI | unk | V | + | + | + | - | - | - | - | C | |  | |  |  |  |  |  |  |  |  |  |  |  |  |
|  | **M013** | MRSA | 2002 | TAI | 437 | Vb | + | + | + | - | - | - | - | C | |  | |  |  |  |  |  |  |  |  |  |  |  |  |
|  | **0864N0066** | MRSA | 1998 | TAI | unk | V | + | + | - | - | - | - | - | X | |  | | + | + |  | + | + | + | + |  | - |  |  | PM9 |
|  | **0864N0093** | MRSA | 2002 | TAI | unk | V | + | + | + | - | - | - | - | C | |  | |  |  |  |  |  |  |  |  |  |  |  |  |
|  | **SA957** | MRSA | 2000 | TAI | 437 | Vb | + | + | + | - | - | - | - | C | |  | |  |  |  |  |  |  |  |  |  |  |  |  |
|  | **0864N0068** | MRSA | 1998 | TAI | unk | V | + | + | + | - | - | - | - | C | |  | |  |  |  |  |  |  |  |  |  |  |  |  |
| **ST59-6** | **0864N0065** | MRSA | 2002 | TAI | unk | IVa | + | + | - | - | - | - | - | X | |  | |  |  |  |  |  |  |  |  |  |  |  |  |
|  | **2245N0074** | MRSA | 2006 | TAI | 437 | IVa | + | + | + | - | - | - | - | C | |  | | + | + | + | + | + | + | + | + | + | + | - | PM1 |
|  | **2245N0081** | MRSA | 2010 | TAI | 437 | IVa | + | + | + | - | - | - | - | C | |  | |  |  |  |  |  |  |  |  |  |  |  |  |
|  | **GD52** | MRSA | 2010 | GD | 437 | IVa | + | + | + | - | - | - | - | C | |  | | + | + | + | + | + | + | + | + | - | + | - | PM9 |
|  | **SA268** | MRSA | 2012 | CHI | 441 | IV | + | + | + | - | - | - | - | C | |  | |  |  |  |  |  |  |  |  |  |  |  |  |
|  | **4297** | MRSA | 2016 | CHI | 437 | IVa | + | + | + | + | + | - | - | C* | |  | | + | + | + | + | + | + | + |  | - |  | - | PM9 |
|  | **705** | MRSA | 2012 | CHI | unk | IVa | + | + | + | + | + | - | - | C* | |  | |  |  |  |  |  |  |  |  |  |  |  |  |
|  | **GD1054** | MRSA | 2010 | GD | 437 | IVa | + | + | + | + | + | - | - | C* | |  | | + | + | + | + | + | + | + | + | - | + | - | PM9 |
|  | **GD27** | MRSA | 2010 | GD | 441 | IVa | + | + | + | + | + | - | - | C* | |  | | + | + | + | + | + | + | + | + | - | + | - | PM9 |
|  | **GD858** | MRSA | 2010 | GD | 4347 | IVa | + | + | + | - | - | - | - | C | |  | | + | + | + | + | + | + | + | + | - | + | - | PM9 |
|  | **0864N0082** | MSSA | 2002 | TAI | 441 | - | - | + | + | - | - | - | - | C | |  | |  |  |  |  |  |  |  |  |  |  |  |  |
|  | **1032** | MSSA | 2012 | CHI | unk | - | - | + | + | + | - | - | + | B | |  | |  | + | + | + | + | + | + |  | + |  | - | PM1 |
|  | **1275** | MSSA | 2012 | CHI | 437 | - | - | + | + | - | - | - | - | C | |  | |  | + | + | + | - | - | - | + | - |  | + | unk-8 |
|  | **1945-0** | MSSA | 2013 | CHI | 1751 | - | - | + | + | - | - | - | - | C | |  | | + | + | + | + | - | - | - |  | - |  | - | unk-7 |
|  | **384** | MSSA | 2012 | CHI | 437 | - | - | + | + | - | - | - | - | C | |  | | + | - | - | - | - | - | - | - | - | - | - | PM18 |
|  | **1827** | MRSA | 2013 | CHI | 437 | IVc | - | + | + | - | - | - | - | C | |  | |  | + | + | + | + | + | + |  | - |  | - | PM9 |
|  | **GD1976** | MRSA | 2010 | GD | 437 | IV | - | + | + | - | - | - | - | C | |  | | + | + | + | + | + | + | + |  | - |  |  | PM9 |
|  | **4226B2** | MSSA | 2016 | CHI | 437 | - | - | + | + | - | - | - | - | C | |  | |  | + | + | + | + | + | + |  | + |  | - | PM1 |
|  | **GD1038** | MRSA | 2010 | GD | 437 | IVg | - | + | + | - | - | - | - | C | |  | | + | + | + | + | + | + | + | + | + | + | - | PM1 |
|  | **GD954** | MSSA | 2010 | GD | 437 | - | - | + | + | - | - | - | - | C | |  | | + | + | + | + | + | + | + | + | - |  | - | PM9 |
|  | **GD1176** | MSSA | 2010 | GD | 437 | - | - | + | + | - | - | - | - | C | |  | | + | - | - | - | - | - | - | - | - | - | - | PM18 |
|  | **GD511** | MSSA | 2010 | GD | 437 | - | + | + | + | - | - | - | - | C | |  | | + | + | + | + | + | + | + | + | - | + | - | PM9 |
|  | **GD1171** | MSSA | 2010 | GD | 8886 | - | - | + | + | - | - | - | - | C | |  | | + | + | + | + | + | + | + | + | + | + | - | PM1 |
|  | **GD843** | MSSA | 2010 | GD | 8886 | - | - | + | + | - | - | - | - | C | |  | | + | - | - | - | - | - | - | - | - | - | - | PM18 |
|  | **GD1605** | MSSA | 2010 | GD | 1751 | - | - | + | + | - | - | - | - | C | |  | | + | + | + | + | + | + | + | + | + | + | - | PM1 |
|  | **GD251** | MSSA | 2010 | GD | 3485 | - | - | + | + | - | - | - | - | C | |  | | + | + | + | + | + | + | + | + | + | + | - | PM1 |
|  | **GD910** | MSSA | 2010 | GD | 4134 | - | - | + | + | - | - | - | - | C | |  | | + | - | - | - | - | - | - | - | - | - | - | PM18 |
|  | **GD1941** | MRSA | 2010 | GD | 437 | IVa | - | + | + | - | - | - | - | C | |  | | + | + | + | + | + | + | + | + | + | + | - | PM1 |
|  | **1956-2** | MSSA | 2013 | CHI | 437 | - | + | + | + | - | - | - | - | C | |  | |  | + | + | + | + | + | + | + | + | + | - | PM1 |
|  | **SA28** | MSSA | 2003 | ACH | 437 | - | + | + | + | - | - | - | - | C | |  | | + | + | + | + | + | + | + | + | - | + | - | PM9 |
|  | **0864N0030** | MSSA | 2006 | UK | unk | - | + | + | - | - | - | - | - | X | |  | |  | + | + | + | + | + | + |  | + |  | - | PM1 |
| **ST59-3** | **0864N0042** | MSSA | 2008 | UK | 441 | - | + | + | + | + | - | - | - | C** | |  | |  |  |  |  |  |  |  |  |  |  |  |  |
|  | **0864N0095** | MRSA | 2010 | UK | unk | IVa | + | + | + | + | - | - | - | C** | |  | |  | + | + | + | + | + | + |  | - |  | - | PM9 |
|  | **2284-0** | MRSA | 2013 | CHI | unk | IVa | - | + | + | - | - | - | - | C | |  | |  |  |  |  |  |  |  |  |  |  |  |  |
|  | **2704-0** | MRSA | 2014 | CHI | 437 | IVa | + | + | + | + | - | - | - | C** | |  | |  | + | + | + | + | + | + |  | - |  | - | PM9 |
|  | **3025** | MRSA | 2015 | CHI | 437 | IVa | + | + | + | + | - | - | + | B | |  | |  |  |  |  |  |  |  |  |  |  |  |  |
|  | **2939** | MRSA | 2015 | CHI | 437 | IVa | - | + | + | - | - | - | - | C | |  | |  | + | + | + | + | + | + |  | - |  | - | PM9 |
|  | **3939** | MRSA | 2016 | CHI | 441 | IVa | - | + | + | - | - | - | - | C | |  | |  |  |  |  |  |  |  |  |  |  |  |  |
|  | **3295** | MRSA | 2015 | CHI | 437 | IVa | + | + | + | + | - | - | - | C** | |  | |  |  |  |  |  |  |  |  |  |  |  |  |
|  | **673** | MRSA | 2012 | CHI | 437 | IVa | + | + | + | - | - | - | - | C | |  | |  |  |  |  |  |  |  |  |  |  |  |  |
|  | **4090A2** | MRSA | 2016 | CHI | 437 | IVa | - | + | + | + | - | - | + | B | |  | |  |  |  |  |  |  |  |  |  |  |  |  |
|  | **3109A3** | MRSA | 2013 | CHI | 437 | IVa | - | + | + | + | - | - | + | B | |  | |  |  |  |  |  |  |  |  |  |  |  |  |
|  | **GD75** | MRSA | 2010 | GD | 437 | IVa | - | + | + | + | - | - | + | B | |  | | + | + | + | + | + | + | + | + | - | + | - | PM9 |
|  | **GD912** | MRSA | 2010 | GD | 437 | IVa | - | + | + | + | - | - | + | B | |  | | + | + | + | + | + | + | + | + | - | + | - | PM9 |
|  | **4127A1** | MRSA | 2016 | CHI | 437 | IVa | - | + | + | + | - | - | + | B | |  | |  |  |  |  |  |  |  |  |  |  |  |  |
|  | **GD81** | MRSA | 2010 | GD | 437 | IVa | - | + | + | + | - | - | + | B | |  | | + | + | + | + | + | + | + | + | - | + | - | PM9 |
|  | **GD19** | MRSA | 2010 | GD | 437 | IVa | - | + | + | + | - | - | + | B | |  | | + | + | + | + | + | + | + | + | - | + | - | PM9 |
|  | **GD517** | MRSA | 2010 | GD | 437 | IVa | - | + | + | + | - | - | + | B | |  | | + | - | - | - | - | - | - | - | - | - | - | PM18 |
|  | **446** | MRSA | 2012 | CHI | 441 | IVa | - | + | + | + | - | - | + | B | |  | | + | + | + | + | + | + | + |  | - |  | - | PM9 |
|  | **GD3.1** | MRSA | 2010 | GD | 441 | IVa | - | + | + | + | - | - | + | B | |  | | + | + | + | + | + | + | + | + | - | + | - | PM9 |
|  | **GD31** | MRSA | 2010 | GD | 441 | IVa | - | + | + | + | - | - | + | B | |  | | + | + | + | + | + | + | + | + | - | + | - | PM9 |
|  | **GD26** | MRSA | 2010 | GD | 437 | IVa | - | + | + | + | - | - | + | B | |  | | + | - | - | - | - | - | - | - | - | - | - | PM18 |
|  | **GD38** | MRSA | 2010 | GD | 437 | IVa | - | + | + | + | - | - | + | B | |  | | + | + | + | + | + | + | + |  | - |  | - | PM9 |
|  | **GD1907** | MRSA | 2010 | GD | 437 | IVa | - | + | + | + | - | - | + | B | |  | | + | + | + | + | + | + | + | + | - | + | - | PM9 |
|  | **L18** | MRSA | 2017 | CHI | 437 | IVa | - | + | + | + | - | - | + | B | |  | |  |  | + | + | + | + | + |  | - |  | - | PM9 |
|  | **GD1958** | MRSA | 2010 | GD | 437 | IVa | - | + | + | + | - | - | + | B | |  | | + | + | + | + | + | + | + | + | - | + | - | PM9 |
|  | **917-0** | MRSA | 2012 | CHI | 441 | IVa | - | - | - | - | - | - | - | - | |  | |  |  |  |  |  |  |  |  |  |  |  |  |
|  | **GD15** | MRSA | 2010 | GD | 437 | IVa | - | + | + | + | - | - | + | B | |  | | + | + | + | + | + | + | + | + | - | + | - | PM9 |
|  | **GD1633** | MRSA | 2010 | GD | 441 | IVa | + | + | + | + | - | - | - | C** | |  | | + | + | + | + | + | + | + | + | - | + | - | PM9 |
| **ST59-4** | **0864N0011** | MRSA | 2000 | UK | unk | IVg | - | + | - | + | - | + | + | G | |  | |  |  |  |  |  |  |  |  |  |  |  |  |
|  | **0864N0062** | MRSA | 2002 | TAI | unk | IVg | - | + | - | + | - | + | + | G | |  | |  |  |  |  |  |  |  |  |  |  |  |  |
|  | **0864N0083** | MRSA | 2002 | TAI | unk | IVg | - | + | - | + | - | + | + | G | |  | |  | + | - | + | - | - | - |  | + |  | - | unk-6 |
|  | **2245N0067** | MRSA | 2004 | TAI | 3424 | IVg | - | + | - | + | - | + | + | G | |  | |  |  |  |  |  |  |  |  |  |  |  |  |
|  | **SA40** | MRSA | 2005 | TAI | 441 | IVg | - | + | - | - | - | - | - | X | |  | |  |  |  |  |  |  |  |  |  |  |  |  |
|  | **0864N0069** | MRSA | 1998 | TAI | 441 | IVg | - | + | - | + | - | - | + | G | |  | | + | - | - | + | + | - | - |  | - |  | + | unk-5 |
|  | **0864N0073** | MRSA | 2000 | TAI | 441 | IVg | - | + | - | + | - | + | + | G | |  | |  |  |  |  |  |  |  |  |  |  |  |  |
|  | **0864N0086** | MSSA | 2002 | TAI | 441 | - | - | + | - | + | - | + | + | G | |  | |  |  |  |  |  |  |  |  |  |  |  |  |
|  | **0864N0070** | MRSA | 1998 | TAI | 441 | IVg | - | + | - | + | - | + | + | G | |  | |  |  |  |  |  |  |  |  |  |  |  |  |
|  | **0864N0075** | MRSA | 2000 | TAI | 437 | IVg | - | + | - | + | - | + | + | G | |  | |  |  |  |  |  |  |  |  |  |  |  |  |
|  | **2245N0062** | MRSA | 2004 | TAI | 437 | IVg | - | + | - | + | - | + | + | G | |  | | + | + | + | + | + | - | - |  | + |  | + | 6272-2 |
|  | **2245N0068** | MRSA | 2004 | TAI | 437 | IVg | - | + | - | + | - | + | + | G | |  | |  |  |  |  |  |  |  |  |  |  |  |  |
|  | **2245N0064** | MRSA | 2004 | TAI | 437 | IVg | - | + | - | + | - | + | + | G | |  | |  |  |  |  |  |  |  |  |  |  |  |  |
|  | **2245N0073** | MRSA | 2006 | TAI | 1751 | IVg | + | + | - | + | - | + | - | G | |  | | + | + | + | + | + | - | - |  | + |  | + | 6272-2 |
|  | **0864N0072** | MRSA | 1998 | TAI | unk | IVg | - | + | - | + | - | + | + | G | |  | |  | + | - | + | + | - | - |  | + |  | + | unk-4 |
|  | **2245N0079** | MRSA | 2008 | TAI | 1751 | IV | - | + | - | + | - | + | + | G | |  | |  | + | + | + | - | - | - |  | + | + | + | unk-3 |
|  | **GD875** | MSSA | 2010 | GD | 1751 | - | - | + | - | + | - | + | + | G | |  | | + | + | + | + | + | + | - | + | + | + | - | unk-1 |
|  | **2574-0** | MRSA | 2004 | CHI | 1751 | IVg | - | + | - | + | - | + | + | G | |  | |  | + | + | + | - | - | - | + | + |  | - | unk-2 |
|  | **0864N0084** | MRSA | 2002 | TAI | 6750 | IVg | - | + | - | + | - | + | + | G | |  | |  |  |  |  |  |  |  |  |  |  |  |  |
|  | **2245N0071** | MRSA | 2006 | TAI | 441 | IVg | - | + | - | + | - | + | + | G | |  | | + | + | + | + | + | - | - |  | + |  | + | 6272-2 |
|  | **2245N0088** | MRSA | 2010 | TAI | 5120 | IVg | - | + | - | + | - | + | + | G | |  | |  |  |  |  |  |  |  |  |  |  |  |  |
|  | **GD1409** | MRSA | 2010 | GD | 437 | IVg | - | + | - | + | - | + | + | G | |  | | + | + | + | + | + | + | - | + | + | + | - | unk-1 |
|  | **0864N0074** | MRSA | 2000 | TAI | unk | IVg | - | + | - | + | - | + | + | G | |  | |  |  |  |  |  |  |  |  |  |  |  |  |
|  | **2245N0084** | MRSA | 2010 | TAI | 437 | IVg | - | + | + | + | - | - | + | B | |  | |  |  |  |  |  |  |  |  |  |  |  |  |
|  | **0864N0071** | MRSA | 1998 | TAI | 441 | IVg | - | + | + | + | - | - | + | B | |  | |  | + | + | + | + | + | + |  | + |  | - | PM1 |
|  | **0864N0067** | MRSA | 1998 | TAI | unk | IVg | - | + | - | + | - | + | + | G | |  | |  |  |  |  |  |  |  |  |  |  |  |  |
|  | **0864N0076** | MRSA | 2000 | TAI | 441 | IVc | - | + | + | + | - | - | + | B | |  | |  | + | + | + | + | + | + |  | + |  | - | PM1 |
|  | **2245N0066** | MRSA | 2004 | TAI | 437 | IVc | - | + | + | + | - | - | + | B | |  | |  | + | + | + | + | + | + |  | + |  | - | PM1 |
|  | **2245N0082** | MRSA | 2010 | TAI | 1151 | IVc | - | + | + | - | - | - | - | C | |  | |  |  |  | - |  |  |  |  |  |  |  | - |
| **ST59-2** | **0864N0001** | MSSA | 2009 | USA | 529 | - | - | + | + | - | - | - | - | C | |  | |  |  |  | - |  |  |  |  |  |  |  | - |
|  | **0864N0005** | MSSA | 2008 | USA | unk | - | - | + | + | + | - | - | + | B | |  | |  |  |  | - |  |  |  |  |  |  |  | - |
|  | **0864N0088** | MSSA | 2005 | USA | unk | - | - | + | + | - | - | - | - | C | |  | |  |  |  | - |  |  |  |  |  |  |  | - |
|  | **0864N0002** | MSSA | 2010 | USA | unk | - | - | + | + | - | - | - | - | C | |  | |  |  |  | - |  |  |  |  |  |  |  | - |
|  | **0864N0004** | MSSA | 2010 | USA | 2365 | - | - | + | + | + | - | - | + | B | |  | |  |  |  | - |  |  |  |  |  |  |  | - |
|  | **0864N0003** | MSSA | 2009 | USA | unk | - | - | + | + | - | - | - | - | C | |  | |  |  |  | - |  |  |  |  |  |  |  | - |
|  | **0864N0008** | MSSA | 2005 | USA | unk | - | - | + | + | + | - | - | + | B | |  | |  |  |  | - |  |  |  |  |  |  |  | - |
|  | **0864N0045** | MRSA | 2010 | UK | unk | IVa | - | + | + | - | - | - | - | C | |  | |  |  |  | - |  |  |  |  |  |  |  | - |
|  | **0864N0010** | MSSA | 2005 | USA | unk | - | - | + | + | - | - | - | - | C | |  | |  |  |  | - |  |  |  |  |  |  |  | - |
|  | **0864N0033** | MSSA | 2010 | UK | unk | - | - | + | + | - | - | - | - | C | |  | |  |  |  | - |  |  |  |  |  |  |  | - |
|  | **290N** | MSSA | 2014 | CAN | 216 | - | - | + | + | - | - | - | - | C | |  | |  |  |  | - | - | - | - |  | - |  | - | - |
|  | **2245N0055** | MSSA | 2010 | USA | 437 | - | - | + | + | - | - | - | - | C | |  | |  |  |  | - |  |  |  |  |  |  |  | - |
|  | **KAE** | MSSA | 2007 | CAN | 2365 | - | - | + | + | - | - | - | - | C | |  | |  |  |  | - | - | - | - |  | - |  | - | - |
|  | **0864N0087** | MSSA | 2010 | USA | 3424 | - | - | + | + | - | - | - | - | C | |  | |  |  |  | - |  |  |  |  |  |  |  | - |
|  | **0864N0046** | MSSA | 2010 | UK | unk | - | - | + | + | + | - | - | + | B | |  | |  |  |  | - |  |  |  |  |  |  |  | - |
|  | **0864N0040** | MSSA | 2010 | UK | 543 | - | - | + | + | + | + | - | - | C* | |  | |  |  |  | - |  |  |  |  |  |  |  | - |
|  | **0864N0096** | MSSA | 2011 | UK | unk | - | - | + | + | + | - | - | + | B | |  | |  |  |  | - |  |  |  |  |  |  |  | - |
|  | **0864N0007** | MSSA | 2004 | USA | unk | - | - | + | + | + | - | - | + | B | |  | |  |  |  | - |  |  |  |  |  |  |  | - |
|  | **0864N0009** | MSSA | 2004 | USA | unk | - | - | + | + | + | + | - | + | B* | |  | |  |  |  | - |  |  |  |  |  |  |  | - |
|  | **0864N0012** | MRSA | 2000 | UK | unk | V | - | + | + | + | - | - | + | B | |  | |  |  |  | - |  |  |  |  |  |  |  | - |
|  | **0864N0013** | MRSA | 2005 | UK | 316 | V | - | + | + | + | - | - | + | B | |  | |  |  |  | - |  |  |  |  |  |  |  | - |
|  | **0864N0044** | MRSA | 2009 | UK | 7344 | V | - | + | + | + | - | - | + | B | |  | |  |  |  | - |  |  |  |  |  |  |  | - |
|  | **0864N0034** | MSSA | 2009 | UK | unk | - | - | + | + | + | - | - | - | C** | |  | |  |  |  | - |  |  |  |  |  |  |  | - |
|  | 0864N0036 | MRSA | 2010 | UK | 316 | V | - | + | + | + | - | - | + | B | |  | |  |  |  | - |  |  |  |  |  |  |  | - |
|  | **0864N0035** | MSSA | 2009 | UK | 316 | - | - | + | + | + | - | - | - | C** | |  | |  |  |  | - |  |  |  |  |  |  |  | - |
|  | **0864N0091** | MSSA | 2005 | USA | unk | - | + | + | + | - | - | - | - | C | |  | |  |  |  | - |  |  |  |  |  |  |  | - |
|  | **SSR** | MSSA | 2012 | CAN | 216 | - | - | + | + | - | - | - | - | C | |  | |  |  |  | - | - | - | - |  | - |  | - | - |
|  | **0864N0006** | MSSA | 2004 | UK | unk | - | - | + | + | + | + | - | + | B* | |  | |  |  |  | - |  |  |  |  |  |  |  | - |
|  | **0864N0090** | MRSA | 2005 | USA | unk | IVg | - | + | + | + | - | + | + | B** | |  | |  |  |  | - |  |  |  |  |  |  |  | - |
| **ST59-1** | **0864N0015** | MSSA | 2011 | UK | 172 | - | + | - | + | - | - | - | - | unk-1 | |  | |  |  |  | - | - | - | - |  | - |  | - | - |
|  | **0864N0041** | MSSA | 2008 | UK | unk | - | - | + | + | + | + | - | - | C* | |  | |  |  |  | - | - | - | - |  | - |  | - | - |
|  | **GD321** | MSSA | 2010 | GD | 163 | - | - | + | + | + | + | - | + | B* | |  | |  |  |  | - | - | - | - |  | - |  | - | - |
|  | **H291** | MRSA | 1992 | CAN | 163 | IV | - | + | + | + | + | - | - | C* | |  | | - | - | - | - | - | - | - | - | - | - | - | - |
|  | **H509** | MSSA | 1993 | CAN | 172 | - | - | + | + | + | + | - | - | C* | |  | | - | - | - | - | - | - | - | - | - | - | - | - |
|  | **PRE** | MSSA | 2001 | CAN | 163 | - | - | + | + | + | + | - | - | C* | |  | | - | - | - | - | - | - | - | - | - | - | - | - |
|  | **S2459** | MSSA | 2010 | CAN | 163 | - | - | + | + | + | + | - | - | C* | |  | |  |  |  | - | - | - | - |  | - |  | - | - |

Note: MRSA, methicillin resistant *Staphylococcus aureus*; MSSA, methicillin sensitive *Staphylococcus aureus*; PFGE, Pulsed field gel electrophoresis; *spa*, staphylococcal protein A; SCC*mec*, Staphylococcal cassette chromosome *mec*; PVL, Panton-Valentine leucocidin; IEC, immune evasion cluster; *scn*, complement inhibitor ; *chp*, chemotaxis-inhibiting protein; *sak*, Staphylokinase precursor; *sea*, staphylococcal enterotoxin A precursor; *sep*, staphylococcal enterotoxin P precursor; MES, mobile element structure; *tnp* tn551, tn551 transposase ; *tnpR*, resolvase; *ermB*, erythromycin resistance transferase; *aph(3')-III*, aminoglycoside 3’-phosphotransferase; *sat*, streptothricin acetyltransferase; *aadE*, streptomycin adenyltransferaser; *cat*, chloramphenicol acetyltransferase; *aacA-aphD*, bifunctional aminoglycoside modifying enzyme; ‡, as defined by Hung et al. {Hung, 2016 #25} and this study; †, as defined by Hung et al. {Hung, 2012 #24} and this study; +, CAN, Canada; CHI, China; GD, Guangdong, China; MAL, Malaysia; NED, Netherlands; TAI, Thailand; UK, United Kingdom; USA, United States of America; positive for trait/gene; -, negative for trait/gene; * and **, as defined by this study; unk, unknown. Data was left blank if not known or determined.
