# Supplementary material for: Staphylococcus aureus ST59: Concurrent but Separate Evolution of North American and East Asian Lineages
Source: Front Microbiol. 2021 Feb 10;12:631845. doi: 10.3389/fmicb.2021.631845 (PMC7902796; doi:10.3389/fmicb.2021.631845)
Supplement: Supplementary file 2 [file Data_Sheet_1.PDF]

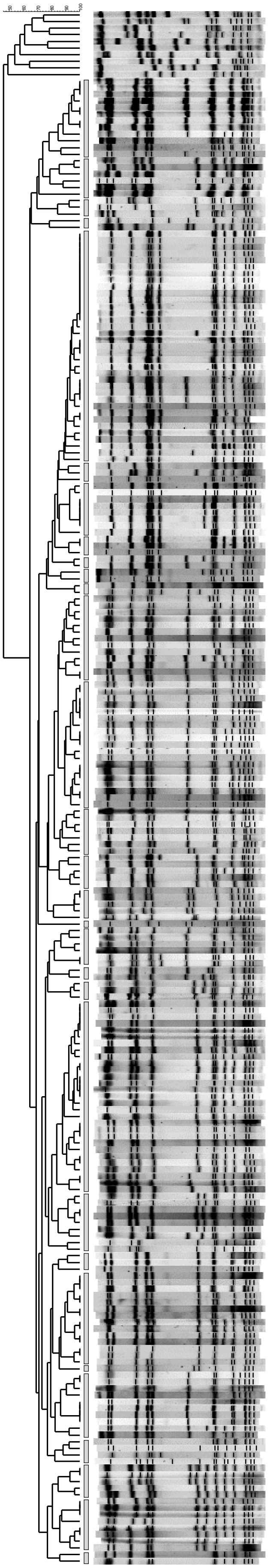

Strain	Country	Year	MRSA/MSSA	*spa* type	SCC*mec* type	PVL	PFGE group (% DCC)
CMRSA10	CAN		MRSA	t008	IVa	+	
CMRSA7	CAN		MRSA	t128	IVa	+	
CMRSA3	CAN		MRSA	t037	IIIHg	-	
CMRSA6	CAN		MRSA	t037	IIIHg	-	
CMRSA-5	CAN		MRSA	t064	IVd	-	
CMRSA9	CAN		MRSA	t008	VIII	-	
CMRSA2	CAN		MRSA	t002	II	-	
CMRSA1	CAN		MRSA	t004	II	-	
CMRSA8	CAN		MRSA	t022	IV	-	
CMRSA4	CAN		MRSA	t018	II	-	
C3347	CAN	2000	MRSA	t437	IV	-	
C5148	CAN	1999	MRSA	t437	IV	-	
C529	CAN	2005	MRSA	t437	IV	-	
C1658	CAN	2006	MRSA	t437	IV	-	
C5732	CAN	2004	MRSA	t437	IV	-	
C4922	CAN	1999	MRSA	t437	IV	-	
C491	CAN	2005	MRSA	t437	IV	-	
C8137	CAN	2005	MRSA	t437	IV	-	
C2407	CAN	2007	MRSA	t437	IV	-	
C440	CAN	2007	MRSA	t437	IV	-	
C16784	CAN	2008	MRSA	t437	IV	-	
GD26	GD	2010	MRSA	t437	IV	-	1 (65%)
GD1594	GD	2010	MSSA	t3220		+	
GD1605	GD	2010	MSSA	t1751		-	
52250675	CAN		MSSA	t163		-	
GD685	GD	2010	MSSA	t3401		-	
H509	CAN	1993	MSSA	t172		-	
C5544	CAN	2000	MRSA	t437	IV	-	
GD827	GD	2010	MSSA	t437		-	
GD843	GD	2010	MSSA	t8886		-	
GD378	GD	2010	MSSA	t437		-	
GD1120.1	GD	2010	MRSA	t437	IV	-	
GD1958	GD	2010	MRSA	t437	IV	-	
C10859	CAN	2006	MRSA	t437	V	+	
GD1196	GD	2010	MRSA	t437	V	+	
GD1197	GD	2010	MRSA	t437	V	+	
GD1199	GD	2010	MRSA	t437	V	+	
GD14	GD	2010	MRSA	t437	V	+	
GD39	GD	2010	MRSA	t437	V	+	
GD51.2	GD	2010	MRSA	t437	V	+	
GD57	GD	2010	MRSA	t3590	V	+	
G097	GEN	2010	MRSA	t437	V	+	
JK3524	CAN	2011	MRSA	t437	V	+	
JK3674	CAN	2011	MRSA	t437	V	+	
CAN20	MAL		MRSA	t3590	Vb	+	
CAN49	MAL		MRSA	t437	V	+	
ST16	MAL		MRSA	t437	V	+	
GD13	GD	2010	MRSA	t437	V	+	
SA28	CAN	2003	MSSA	t437		+	
C7883	CAN	2004	MRSA	t437	IIIHg	+	
GD1195	GD	2010	MRSA	t437	V	+	
GD59.1	GD	2010	MRSA	t437	V	+	
JK3997	CAN	2012	MRSA	t437	V	+	
C3714	CAN	2006	MRSA	new	Vb	+	
GD1572	GD	2010	MSSA	t437		-	
GD1965	GD	2010	MRSA	t437	IV	-	
GD858	GD	2010	MRSA	t437	IVa	+	
GD859	GD	2010	MRSA	t437	IVa	+	
GD862	GD	2010	MRSA	t437	IVa	+	
GD65	GD	2010	MRSA	t3736	unk	+	
GD62	GD	2010	MRSA	t437	V	+	2 (76%)
GD74	GD	2010	MRSA	t437	V	-	
GD21	GD	2010	MRSA	t437	V	+	
C4978	CAN	2001	MRSA	t437	V	+	
GD18	GD	2010	MRSA	t437	V	+	
C479	CAN	2006	MRSA	t437	Vb	-	
GD7	GD	2010	MRSA	t3590	V	+	
GD48	GD	2010	MRSA	t437	V	+	
GD1690	GD	2010	MRSA	t437	V	+	
GD511	GD	2010	MSSA	t437		+	
GD29	GD	2010	MRSA	t437	V	+	
C17335	CAN	2007	MRSA	t693	V	+	
GD1977	GD	2010	MRSA	t693	V	+	
C6242	CAN	2007	MRSA	t437	V	+	
C889b	CAN	2008	MRSA	t437	Vb	-	
C16703	CAN	2008	MRSA	t437	V	-	
GD1850	GD	2010	MRSA	t437	unk	-	
GD1883	GD	2010	MRSA	t437	unk	+	
C6539	CAN	2007	MRSA	t437	V	+	
C11224	CAN	2006	MRSA	t437	Vb	+	
C17841	CAN	2008	MRSA	t437	V	+	
GD30	GD	2010	MRSA	t437	V	+	
GD52	GD	2010	MRSA	t437	IVa	+	
GD876	GD	2010	MRSA	t7281	IVa	+	
M07MS-625	CAN	2007	MRSA	t437	Vb	-	
M08MS-60	CAN	2008	MRSA	t437	Vb	-	
GD1633	GD	2010	MRSA	t441	IVa	-	
GD68	GD	2010	MRSA	t437	IVa	+	
GD1514	GD	2010	MSSA	t437		-	
GD66	GD	2010	MRSA	t437	IVa	-	
GD1112	GD	2010	MRSA	t437	IV	-	
GD81	GD	2010	MRSA	t437	IVa	-	
GD45	GD	2010	MRSA	t437	IVa	-	
GD883	GD	2010	MRSA	t1751	IV	-	
GD1360	GD	2010	MRSA	t3523	IV	-	
GD70	GD	2010	MRSA	t437	IV	-	
GD1208	GD	2010	MRSA	t437	IV	-	
GD1038	GD	2010	MRSA	t437	IV	-	
GD878	GD	2010	MRSA	t437	IVa	-	
GD82	GD	2010	MRSA	t3485	IVa	-	
GD874	GD	2010	MRSA	t3485	IVa	-	
GD1982	GD	2010	MRSA	t437	IV	-	
GD518	GD	2010	MRSA	t437	IV	-	
GD15	GD	2010	MRSA	t437	IVa	-	
GD784	GD	2010	MRSA	t437	IVa	-	
GD786	GD	2010	MRSA	t437	IVa	-	
GD89	GD	2010	MRSA	t437	IVa	-	
GD1086	GD	2010	MRSA	t3485	IV	-	
GD103	GD	2010	MRSA	t437	IVa	-	
GD38	GD	2010	MRSA	t437	IVa	-	
GD49	GD	2010	MRSA	t437	IVa	-	
C4386	CAN	2005	MRSA	t437	IVa	-	
GD19	GD	2010	MRSA	t437	IVa	-	3 (76%)
GD1563	GD	2010	MSSA	t437		-	
GD1961	GD	2010	MRSA	t437	IV	-	
GD520	GD	2010	MRSA	t437	IVa	-	
GD46	GD	2010	MRSA	t437	IVa	-	
GD1420	GD	2010	MRSA	t437	IV	-	
GD1482	GD	2010	MRSA	t437	IV	-	
GD31	GD	2010	MRSA	t441	IVa	-	
GD1737	GD	2010	MSSA	t091		-	
GD1907	GD	2010	MRSA	t437	IV	-	
C8779	CAN	2005	MRSA	t437	IVa	-	
GD16	GD	2010	MRSA	t437	IVa	-	
GD72	GD	2010	MRSA	t437	IVa	-	
GD1409	GD	2010	MRSA	t437	IV	-	
GD87	GD	2010	MRSA	t437	IV	-	
C7096	CAN	2003	MSSA	t437		+	
GD875	GD	2010	MSSA	t1751		-	
GD1935	GD	2010	MSSA	t437		-	
GD515	GD	2010	MSSA	t441		-	
GD76	GD	2010	MSSA	t437	IVa	-	
GD1946	GD	2010	MRSA	t437	IV	-	
GD1950	GD	2010	MRSA	t437	IV	-	
GD75	GD	2010	MRSA	t437	IVa	-	
GD912	GD	2010	MRSA	t437	IVa	-	
GD1074	GD	2010	MSSA	t437		-	
GD27	GD	2010	MRSA	t441	IVa	-	4
GD3.1	GD	2010	MRSA	t441	IVa	-	
GD886	GD	2010	MRSA	t437	IVa	-	
GD1102	GD	2010	MRSA	t437	IVa	-	
GD1571	GD	2010	MSSA	t437		-	
GD1054	GD	2010	MRSA	t437	IVa	+	
GD872	GD	2010	MRSA	t437	IVa	+	
GD1157	GD	2010	MRSA	t437	IV	-	
GD1941	GD	2010	MRSA	t437	IVa	-	
GD113	GD	2010	MRSA	t437	unk	-	
GD1976	GD	2010	MRSA	t437	unk	-	
GD1153	GD	2010	MSSA	t437		-	
GD1099	GD	2010	MSSA	t437		-	
GD11	GD	2010	MSSA	t437		-	
GD1117	GD	2010	MSSA	new		-	
GD1466	GD	2010	MSSA	t437		-	
GD1851	GD	2010	MRSA	t437	V	+	
GD1848	GD	2010	MRSA	t437	IV	-	
GD910	GD	2010	MSSA	t4134		-	
GD1277	GD	2010	MSSA	t437		-	
GD170	GD	2010	MSSA	t437		-	
GD213	GD	2010	MSSA	t437		-	
GD396	GD	2010	MSSA	t437		-	
GD1091	GD	2010	MSSA	t437		-	
GD1129	GD	2010	MSSA	new		-	
GD1476	GD	2010	MSSA	t324		-	5 (75%)
GD388	GD	2010	MSSA	t437		-	
GD184	GD	2010	MSSA	t437		-	
GD251	GD	2010	MSSA	t3485		-	
GD210	GD	2010	MSSA	t437		-	
GD1421	GD	2010	MSSA	t441		-	
GD507	GD	2010	MSSA	t437		-	
GD546	GD	2010	MSSA	t437		-	
GD1531	GD	2010	MSSA	t441		-	
GD1631	GD	2010	MSSA	t437		-	
GD1603	GD	2010	MSSA	t7960		-	
GD439	GD	2010	MSSA	t437		-	
GD1904	GD	2010	MSSA	t437		-	
GD1217	GD	2010	MSSA	t437		-	
GD1832	GD	2010	MSSA	t091		-	
GD504	GD	2010	MSSA	t437		-	
GD1622	GD	2010	MSSA	t437		-	
GD474	GD	2010	MSSA	t437		-	
GD1171	GD	2010	MSSA	t8886		-	
GD1338	GD	2010	MSSA	t9460		-	
GD1607	GD	2010	MSSA	t701		-	
GD1138	GD	2010	MSSA	t437		-	
GD880	GD	2010	MRSA	t437	IVa	-	
GD1648	GD	2010	MSSA	t437		-	
GD1660	GD	2010	MSSA	t437		-	
C389	GD	2010	MSSA	t163		-	
S2459	CAN	2010	MSSA	t163		-	
C11909	CAN	2003	MSSA	t163		-	
GD1432	GD	2010	MSSA	t163		-	
GD1818	GD	2010	MSSA	t163		-	
H494	CAN	1993	MSSA	t163		-	
KAE	CAN	2007	MSSA	t2365		-	
KAF	CAN	2006	MSSA	t2365		-	
C50678	CAN	2010	MSSA	t1151		-	
C50679	CAN	2010	MSSA	t1151		-	
GD460	GD	2010	MSSA	t216		-	
290N	CAN	2014	MSSA	t216		-	
C2286	CAN	2003	MSSA	t216		-	
C32924	CAN	2011	MSSA	t1151		-	
PRE	CAN	2001	MSSA	t163		-	
PRR	CAN	2004	MSSA	t163		-	6 (76%)
C1019	CAN	2002	MSSA	new		-	
GD321	GD	2010	MSSA	t163		-	
GD41	GD	2010	MRSA	t437	IVa	-	
GD43	GD	2010	MRSA	t437	IVa	-	
GD517	GD	2010	MRSA	t437	IVa	-	
GD519	GD	2010	MRSA	t437	IVa	-	
C10767	CAN	2006	MRSA	t437	IVa	-	
C9677	CAN	2006	MRSA	t437	IVa	-	
C4952	CAN	2005	MRSA	t437	IVa	-	
C9554	CAN	2005	MRSA	t437	IVa	-	
C138262	CAN	2005	MRSA	t437	IVa	-	
H291	CAN	1992	MRSA	t163	IV	-	
C8879	CAN	2007	MRSA	t437	IVa	-	
SSR	CAN	2012	MSSA	t13447		-	
GD84.2	GD	2010	MRSA	t437	V	+	
TWF	CAN	2007	MSSA	t216		-	
GD1325	GD	2010	MSSA	t437		-	
GD954	GD	2010	MSSA	t437		-	
GD945	GD	2010	MSSA	t437		-	
GD272	GD	2010	MSSA	t437		-	
GD427	GD	2010	MSSA	t437		-	
GD124	GD	2010	MSSA	t437		-	
GD1829	GD	2010	MSSA	t091		-	
GD1847	GD	2010	MRSA	t437	IV	-	7 (76%)
GD1176	GD	2010	MSSA	t437		-	
GD1005	GD	2010	MSSA	t437		-	
GD1652	GD	2010	MSSA	t437		-	
GD1887	GD	2010	MSSA	t091		-	
GD896	GD	2010	MSSA	t437		-	
GD1068	GD	2010	MRSA	t693	V	+	
GD1105	GD	2010	MRSA	t437	V	-	

Supplementary Figure 1. Pulsed Field Gel Electrophoresis fingerprints for the ST59 collection. 224 ST59 isolates were identified in our collection and their *smaI* digestion fingerprints and corresponding dendrogram are shown. Country and year of isolation, methicillin resistance pattern, *spa* type, SCC*mec* type and presence/absence of PVL are included. The lineage was divided into 7 PFGE groups, with the percentage similarity of strains in the sub-group (DCC) shown in brackets. Isolates selected for whole genome sequencing are indicated by red font. MSSA are indicated by blue font. Details are left blank if the information is not known. Canadian MRSA epidemic reference strains, CMRSA1-10, were included. *spa*, staphylococcal protein A; MRSA, methicillin resistant *Staphylococcus aureus*; MSSA, methicillin sensitive *Staphylococcus aureus*; SCC*mec*, Staphylococcal cassette chromosome *mec*; PVL, Panton-Valentine leucocidin; CAN, Canada; GD, Guangdong, China; MAL, Malaysia; GEN, Geneva; new, *spa* type not previously recorded; unk, type could not be determined; +, PVL is present; -, PVL is absent.
